# A fine-tuned convolutional neural network model for accurate Alzheimer’s disease classification

**DOI:** 10.1038/s41598-025-86635-2

**Published:** 2025-04-04

**Authors:** Muhammad Zahid Hussain, Tariq Shahzad, Shahid Mehmood, Kainat Akram, Muhammad Adnan Khan, Muhammad Usman Tariq, Arfan Ahmed

**Affiliations:** 1 Faculty of Information Technology and Computer Science, University of Central Punjab, Lahore, 54000 Pakistan; 2 Department of Computer Engineering, COMSATS University Islamabad, Sahiwal Campus, Sahiwal, 57000 Pakistan; 3 Department of Computer Science, Bahria University Lahore Campus, Lahore, 54000 Pakistan; 4 Department of Computer Science, University of Engineering and Technology, Lahore, 54000 Pakistan; 5 Department of Software, Faculty of Artificial Intelligence and Software, Gachon University, Seongnam-si, 13120 Republic of Korea; 6 Abu Dhabi University, Abu Dhabi, UAE; 7 AI Center for Precision Health, Weill Cornell Medicine-Qatar, Doha, Qatar

**Keywords:** Classification, Convolutional neural networks (CNN), Solvers/optimizers, Alzheimer’s disease detection, Transfer learning, Diseases, Health care, Mathematics and computing, Computer science, Machine learning

## Abstract

Alzheimer’s disease (AD) is one of the primary causes of dementia in the older population, affecting memories, cognitive levels, and the ability to accomplish simple activities gradually. Timely intervention and efficient control of the disease prove to be possible through early diagnosis. The conventional machine learning models designed for AD detection work well only up to a certain point. They usually require a lot of labeled data and do not transfer well to new datasets. Additionally, they incur long periods of retraining. Relatively powerful models of deep learning, however, also are very demanding in computational resources and data. In light of these, we put forward a new way of diagnosing AD using magnetic resonance imaging (MRI) scans and transfer learned convolutional neural networks (CNN). Transfer learning makes it easier to reduce the costs involved in training and improves performance because it allows the use of models which have been trained previously and which generalize very well even when there is very little training data available. In this research, we used three different pre-trained CNN based architectures (AlexNet, GoogleNet, and MobileNetV2) each implemented with several solvers (e.g. Adam, Stochastic Gradient Descent or SGD, and Root Mean Square Propagation or RMSprop). Our model achieved impressive classification results of 99.4% on the Kaggle MRI dataset as well as 98.2% on the Open Access Series of Imaging Studies (OASIS) database. Such results serve to demonstrate how transfer learning is an effective solution to the issues related to conventional models that limits the accuracy of diagnosis of AD, thus enabling their earlier and more accurate diagnosis. This would in turn benefit the patients by improving the treatment management and providing insights on the disease progression.

**Supplementary Information:**

The online version contains supplementary material available at 10.1038/s41598-025-86635-2.

## Introduction

Alzheimer’s disease (AD) is a type of dementia that primarily affects older adults, causing significant brain tissue damage and the death of nerve cells. This leads to memory loss and difficulties with daily activities such as reading, writing, speaking, and recognizing family members^1^. In advanced stages, patients may struggle to recognize loved ones and experience severe symptoms like heart failure and respiratory dysfunction, which can be fatal^2^. The symptoms of AD gradually worsen over time, severely impacting overall health^3^. By 2050, it is estimated that one in every 85 individuals will suffer from AD, underscoring its increasing prevalence^4^. Around 60 to 80% of AD patients exhibit dementia symptoms, making it the world’s second most severe neurological disorder^5^. The disease causes the cerebral cortex and hippocampus to shrink while the brain’s ventricles enlarge. The reduction in hippocampal size impairs episodic and spatial memory, leading to problems with judgment, short-term memory, and planning^6^.

Machine learning and deep learning algorithms have proven highly effective in diagnosing a wide range of diseases, including those affecting the heart, lungs, brain, retina, breast, and bones^7–12^. These advanced methods play a vital role in medical image classification, a key area of computational intelligence^13–16^. Convolutional neural networks (CNNs) stand out in deep learning due to their exceptional accuracy in medical image classification. A significant advantage of CNNs over traditional machine learning techniques is their ability to automatically extract the most relevant features and classify the stages of Alzheimer’s disease, eliminating the need for manual feature extraction^17^. Nevertheless, several such works still reported accuracy, generalizability, and data efficiency issues when using MRI data for Alzheimer’s classification. The high dimensionality of MRI data makeup makes the features interdependent and hence there is needed for models which can extract useful features with very little manual effort. There are several studies which utilized MRI data and different AI techniques for the classification glioma, meningioma, and pituitary tumors^18–21^ Several recent studies have examined the reliability of MRI for diagnosing Alzheimer’s disease. For instance, Zhang et al.^22^ proposed a hybrid machine learning approach that requires retraining when new data is introduced, which limits its scalability in clinical applications. Similarly, Liu et al.^23^ explored the use of auto-encoders for classifying Alzheimer’s stages. While the results were promising, the method required significant data preprocessing, which can be resource intensive. Additionally, Bilfaqih et al.^24^ developed a hybrid deep learning approach that achieved 98.5% accuracy using a combination of MRI and other imaging modalities, but the method was constrained by its binary classification output, limiting its broader applicability in distinguishing between multiple stages of Alzheimer’s disease. Our study builds upon the advancements of those prior works in that we are able to diagnose fast and accurately thanks to transfer learning using pre-trained CNNs even for datasets that are relatively small for Alzheimer’s recognition. In this regard, this study is developed as a means of improving the diagnosis of Alzheimer’s disease through the application of transfer learning to counter the challenges posed by scanty labeled data and excessive computational power required to train appropriate models in the medical imaging field.

This study focuses on the early classification of brain tumors using magnetic resonance imaging (MRI) and artificial intelligence (AI) through deep learning (DL). The researchers developed an efficient method based on transfer learning, using pre-trained models such as Xception, NasNet Large, DenseNet121, and InceptionResNetV2 to extract deep features from MRI scans. The study utilized two benchmark datasets, with preprocessing and data augmentation applied to enhance training. The models were trained using three optimizers (ADAM, SGD, RMSprop), and the Xception model with ADAM achieved the best performance, showing 99.67% accuracy on the larger dataset and 91.94% accuracy on the smaller dataset, outperforming other approaches in the field. Our key contributions include:

- Use of transfer learning for MRI-based Alzheimer’s detection: we utilized three pre-trained deep CNNs—AlexNet, GoogLeNet, and MobileNetV2—applying transfer learning to leverage their feature extraction capabilities while minimizing the need for large amounts of labeled training data.
- Comprehensive performance evaluation: we conducted a detailed performance analysis, assessing the effects of different solvers (Adam, Stochastic Gradient Descent, RMSprop) on model fine-tuning and classification accuracy.
- Comparison of pre-trained architectures: we compared these models in terms of accuracy, precision, and recall to determine the best-performing architecture for Alzheimer’s diagnosis using MRI datasets. Our analysis offers valuable insights into the strengths and weaknesses of each model in the context of medical image classification.

The rest of the paper is structured as follows: Sect “Materials and methods” details the dataset we employed and the pre-processing techniques we applied. Sect “Results”offers a brief overview of previous studies. Sect “Limitations” discusses the proposed model and research methods. Sect “Advantages” presents the experimental results and discussions, followed by the conclusion.

### Related works

In the last decade, the medical community has notably advanced imaging techniques such as Positron Emission Tomography (PET), Diffusion Tensor Imaging (DTI), and particularly MRI, which has become a principal method for predicting AD^25^. For the classification and diagnosis of AD, various deep learning techniques, such as support vector machines (SVM) using weighted MRI images and random forest classifiers, have been used^26^. El-Dahshan et al.^27^ suggested a hybrid approach involving feature extraction, classification, and dimensionality reduction, three-step classification techniques. This method is advantageous due to its speed, simplicity, affordability, and non-invasive nature. However, a significant limitation of this hybrid technique is the requirement for new training whenever the dataset is increased. Ahmed et al.^28^ introduced a CNN-based and patch-based classifier for classifying AD, reducing computing costs and significantly advancing the field. This approach involves multiple operations on the dataset, enabling direct disease detection from the input data. In another study, Hong et al.^29^ developed a specialized recurrent neural network (RNN) based on long short-term memory (LSTM) for the identification and classification of AD. This model uses current and historical patient data, making it crucial for timely disease identification.

Furthermore, it primarily focuses on predicting disease progression using time series data rather than just classification, making it more effective in anticipating future symptoms and enabling earlier interventions for Alzheimer’s patients. This method uses the patient’s previous data that are connected to the patient’s present and historical data; this technique is significant for the timely identification of the disease. The proposed technique primarily uses time series data and describes disease prediction instead of classification. Gupta et al.^30^ developed a model for classifying AD using the ADNI dataset. Based on a sparse auto-encoder, the model achieves a maximum accuracy of 95%. Suk et al.^31^ introduced a new approach for classifying stages of AD and Mild Cognitive Impairment (MCI) converters using an auto-encoder network, which achieved an accuracy of 95.9% across these MCI stages. Islam et al.^32^ proposed a CNN-based model for early disease identification built on the Inception-V4 network. The input and output layers were processed using the inception-A, B, and C components and the reduction-A and B components, with input and output passing through numerous filter concatenation procedures. The model achieved a total accuracy of 73.75% during the training and testing phases using the open-access imaging studies (OASIS) database. A feature extraction framework was presented by Guerrero et al.^33^ that was established using substantial inter-subject variability. The proposed model achieved an overall accuracy of 71%. However, it has some limitations due to its implementation as a binary classification model. Gorji et al.^34^ developed a convolutional neural network model with a 94.54% accuracy rate in classifying early and late stages of mild cognitive impairment. In Hosseini-Asl^35^, deep learning techniques have recently solved the inability of many computer-aided diagnosis structures to extrapolate the distinguishing characteristics from the raw picture data automatically. There are four essential phases: features are extracted, segments are created, skulls are stripped, and normalization is required in end-to-end learning to create an accurate prediction of diseases.

Additionally, He et al.^36^ proposed the Deep Residual Network to counteract the erosion of training accuracy, a common challenge exacerbated by the scarcity of annotated datasets. This limitation makes applying CNN in medical imaging particularly challenging, as they require substantial data for practical training. Hazarika et al.^37^ examined several prominent deep-learning models and their effectiveness in classifying AD. Of these, the DenseNet-121 model delivered the best performance. In another study, Shanmugam et al.^38^ tested three pre-trained CNN models (including GoogLeNet, AlexNet, and ResNet-18) for the early detection of AD and related cognitive issues. Among them, ResNet-18 achieved the highest accuracy, recording a rate of 97.51%. Meanwhile, Battineni et al.^39^ utilized various deep neural networks to classify MRI brain images, employing standard and fine-tuned features. This approach led to the highest accuracies of 95.7% with the CNN model and 95.5% with the DenseNet model.

Detecting Alzheimer’s disease (AD) through deep learning techniques has shown promise; however, several challenges persist that hinder the effectiveness and generalizability of these models according to Table 1. Below is an analysis of how our research addresses the gaps in Alzheimer’s detection using MRI images through deep learning:

Computational Resources and Efficiency: Training deep architectures imposes significant computational resources and time overheads. A CNN-based patch classifier designed by Ahmed et al.,^28^ addresses these limitations with respect to computational load, but the ensuing operations related to dataset’s creation are still elaborate and costly in resources, warranting the quest for more effective solutions.

**Table 1:** Previous models with identified limitations.

Study/authors	Method/model	Limitations
El-Dahshan et al.^27^	Hybrid approach with feature extraction, classification, and dimensionality reduction	Requires retraining when the dataset increases, limiting scalability.
Ahmed et al.^28^	CNN-based patch classifier	Computing cost reduced, but dataset operations are complex and resource-intensive.
Hong et al.^29^	RNN based on LSTM for disease progression prediction	Focuses more on time-series prediction than classification; performance depends on availability of historical patient data.
Gupta et al.^30^	Sparse auto-encoder using ADNI dataset	Maximum accuracy of 95%, but no discussion on performance with more challenging datasets or generalization to other domains.
Suk et al.^31^	Auto-encoder network for AD and MCI converters	Achieves 95.9% accuracy, but only for specific stages of MCI, lacking insight into general AD classification.
Islam et al.^32^	CNN-based model built on Inception-V4 network	Lower accuracy of 73.75% on the OASIS dataset; potential underperformance in clinical application due to complexity of the network.
Guerrero et al.^33^	Feature extraction framework	Achieves 71% accuracy; binary classification limits application to more nuanced diagnosis.
Gorji et al.^34^	CNN model for classifying early and late stages of mild cognitive impairment	Accuracy of 94.54% but lacks adaptability to non-MCI Alzheimer’s stages.
Hosseini-As^35^	End-to-end deep learning model for diagnosis	Requires multiple preprocessing steps (feature extraction, skull stripping, normalization), adding complexity and possible error points.
He et al.^36^	Deep Residual Network	Faces training challenges due to the scarcity of annotated data; CNNs need large datasets, which are often unavailable in medical imaging.
Hazarika et al.^37^	DenseNet-121 for AD classification	No major limitations mentioned, but lacks detailed comparison with other architectures for specific Alzheimer’s-related features.
Shanmugam et al.^38^	Pre-trained CNNs (GoogLeNet, AlexNet, ResNet-18)	ResNet-18 achieved the highest accuracy (97.51%); however, other models performed less effectively, suggesting dependency on the model architecture used.
Battineni et al.^30^	CNN and DenseNet models	Accuracy of 95.7% (CNN) and 95.5% (DenseNet); the reliance on fine-tuning for high accuracy may limit generalizability across different MRI datasets.

Our approach addresses the challenge of computational burden by adopting transfer learning, which precludes the training of any models from scratch especially incurring low computational costs. Through this strategy, it also becomes possible to make use of optimized/ trained CNN models which is an improvement from the previous work which were resource-intensive and involved complicated processes. Additionally, we conducted a comparative analysis of three solvers (ADAM, SGD, and RMSprop), providing a comprehensive evaluation of their efficiency in terms of training convergence and performance. This not only optimized model performance but also highlighted the most resource-efficient approaches, making our model more practical for real-world applications where computational resources may be limited.

Early detection and disease progression: while some approaches are devised that focus solely on identifying and classifying stages of the disease after ample brain degeneration has taken place, early-stage detection is quite problematic. For the purpose of disease progression prediction, Hong and colleagues^29^ employed a long-short-term-memory (LSTM)-based recurrent neural network, stressing this method’s scope was largely dependent on historical patient data made available, which can be a limitation for early diagnosis.

Our research effectively addresses the issue of the early detection of different stages of AD, including Very Mild-Demented and Mild-Demented ones. In other words, the model can identify the early phase of dementia, which is essential to start intervention and treatment as soon as possible. This early detection is important as the progression of the disease can be slowed down and the patient’s condition is likely to improve. By focusing on these early stages, our approach fills a key research gap, as identified in the work of Hong et al.,^29^ and demonstrates the model’s capacity to support early-stage dementia identification, where interventions are most effective.

Model Complexity and Interpretability: Accurate predictions have been recorded in performing specific tasks using advanced models such as deep residual networks and autoencoders. However, such advanced models may present problems with regards to interpretation and reproducibility. For example, Hosseini-Asl et al.^35^ proposed an end‐to‐end deep learning system for AD diagnosis but stressed that many preprocessing steps were necessary adding to the complexity and potential sources of errors.

In this research, we used CNN architectures like AlexNet, MobileNetV2, and GoogleNet that strike a good balance between performance and interpretability as opposed to using complex models like autoencoders or deep residual networks. There is a degree of explainability which is intrinsic to CNNs as their convolutional layers are looking at local features like edges or textures of MRI images and make it possible to see which areas of the brain contribute to the model. There is a high importance of this form of interpretability in medical cases, as doctors need to know how the AI is making decisions. Thus, by applying pre-trained networks using transfer learning, we reduced computational complexity while ensuring the model is simple and easy to understand.

Limited dataset size and quality: deep learning architectures like convolutional neural networks (CNNs) rely on large, annotated datasets to achieve their intended purpose effectively. Acquisition of such datasets is difficult in medical imaging due to the lack of labeled data as well as differences in imaging protocols from one institution to the other. For example, He et al.^36^ pointed out that the availability of annotated data is a major bottleneck in training deep networks for AD detection, especially so given that CNNs more often require large datasets.

In our study, we efficiently incorporate transfer learning by incorporating pre-trained models (AlexNet, MobileNetV2, and GoogleNet), which relieves the burden of large scale, well-annotated datasets. Datasets that are smaller in size can be used to fine-tune pre-trained models to address the challenge of dataset scarcity. Moreover, the application of transfer learning in medical image analysis has gained much acceptance to address the challenge of limited dataset size.

Generalization across datasets: a model that is trained on specific data may not work as effectively on other data sets due to its unique distributions, imaging methods, or even different patients. For instance, Battineni et al.^39^ achieved accurate results employing CNN and DenseNet architecture for Advanced Dementia diagnosis but indicated that some level of fine-tuning is required to achieve such results efficiently, may limit generalizability across different MRI datasets.

This work displays great results for the classification of AD with MRI images both on the Kaggle dataset as well as OASIS. Nonetheless, we consider assessment of the model’s generalizability as very important, and we agree with the opinion of other studies, such as the one conducted by Battineni et al.,^39^. Therefore, further research intends to incorporate external validation on different datasets to evaluate the performance of the model in various clinical and imaging conditions.

## Materials and methods

### Employed datasets

**Fig. 1:**
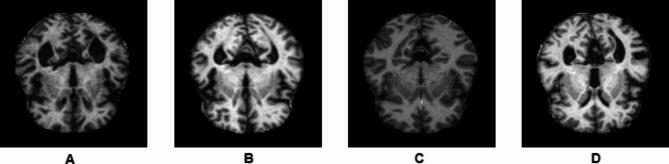
Sample MRI images of the brain from the first dataset, used to test our proposed model for identifying and classifying Alzheimer’s disease (AD). The MRI images include: (**A**) mild demented, (**B**) moderate demented, (**C**) non-demented, and (**D**) very mild demented.

Using two MRI datasets, we tested our proposed model for identifying and classifying AD. The first dataset, available on Kaggle^40^ and referred to here as the Kaggle dataset, is a multi-class, multi-label database containing 5000 images divided into four classes, each representing different stages of dementia severity (Table 2). Given that the original images in the Kaggle dataset were highly imbalanced, which could lead to overfitting issues during deep learning training, we increased the number of images. Different techniques were used to augment the data and improve model performance. Vertical and horizontal flipping, along with rotation, were applied to enhance the diversity of the dataset. Initially, images were rotated by 45° to introduce variation. If class imbalance remained, additional rotations of 90 and 180° were performed. These combined techniques ensured a balanced number of images across all classes, helping the model generalize more effectively. For the augmentation process, the same techniques were applied uniformly across all classes to maintain consistency. This approach successfully expanded the Kaggle dataset to 10,254 MRI images (Table 2; Fig. 1). Figure 1 shows sample images for each class from the Alzheimer’s disease dataset. The images were resized to meet the input specifications of the pre-trained models.

**Table 2:** Class-wise details of AD Kaggle dataset^40^.

Sr. #	Class	Total no of images
1	Mild-demented	2561
2	Moderate-demented	2570
3	Non-demented	2560
4	Very mild-demented	2563

We also performed the external evaluation of our proposed model using a second dataset of 382 MRI images sourced from the OASIS database curated by Daniel S. Marcus at the Neuroimaging Informatics Analysis Center (NIAC) at Washington University School of Medicine^41^ (Table 3). This dataset, referred to here as the OASIS dataset, consists of 3 to 4 individual T1-weighted MRI scans contributed by each subject, all obtained during a single imaging session. For classification purposes, we divided the images into four classes according to the (CDR) scores: CDR-0 (non-demented), CDR-0.5 (very mild-demented), CDR-1 (mild-demented), and CDR-2 (moderate-demented).

**Table 3:** Class-wise details of MRI images sourced from the OASIS database^41^.

Sr. #	Class	Original number of images	Total no. of images after augmentation
1	Mild-demented	105	840
2	Moderate-demented	23	460
3	Non-demented	167	835
4	Very mild-demented	87	609

This dataset comprises MRI images of AD patients aged 23 to 86 years. To ensure balanced data and enhance the model’s learning efficacy, we also applied an augmentation strategy to this dataset. While the original OASIS^41^ dataset images are 256 × 256 pixels, our proposed CNN model requires images to be resized to 224 × 224 pixels. Therefore, we appropriately scaled the OASIS dataset images to fit the required dimensions for our model. Finally, for both datasets, we partitioned the data into two subsets: the training set, which comprises 80% of the images, and the test set, which contains the remaining 20% to provide sufficient training data, ensure better generalization, and balance overfitting and underfitting. Although the Kaggle dataset is popular, it had problems with class imbalance and a limited number of images, which were fixed during model training. We evaluated the trained model’s generalizability using the OASIS dataset for external validation, and it performed admirably. Our study is currently restricted to these two datasets, but we intend to test the models on more datasets in the future to assess their generalizability and performance in further detail.

**Fig. 2:**
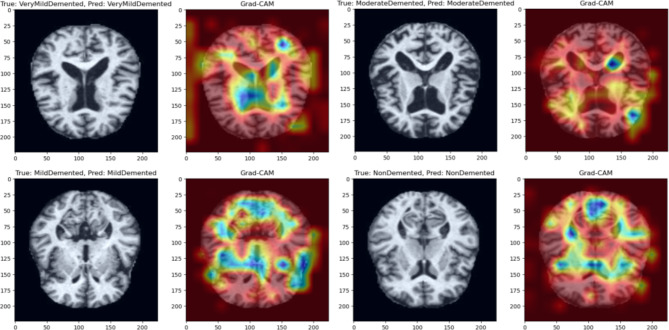
Grad-CAM visualizations of correctly MRIs.

### Grad-CAM visualizations

Figure 2 presents the Grad-CAM Visualizations of Correctly MRIs. Original MR images of four different types alongside their respective Grad-CAM heatmaps. Each pair shows the original image on the left, followed by the Grad-CAM visualization on the right. The true and predicted classes are displayed for each image. Grad-CAM highlights the regions of the image that the model focused on when making its classification decision. Warmer colors (red/yellow) indicate regions with a higher contribution to the model’s decision, while cooler colors (blue/green) signify less impact. These visualizations provide insight into the model’s attention to key features that align with expert-based classification criteria.

**Fig. 3:**
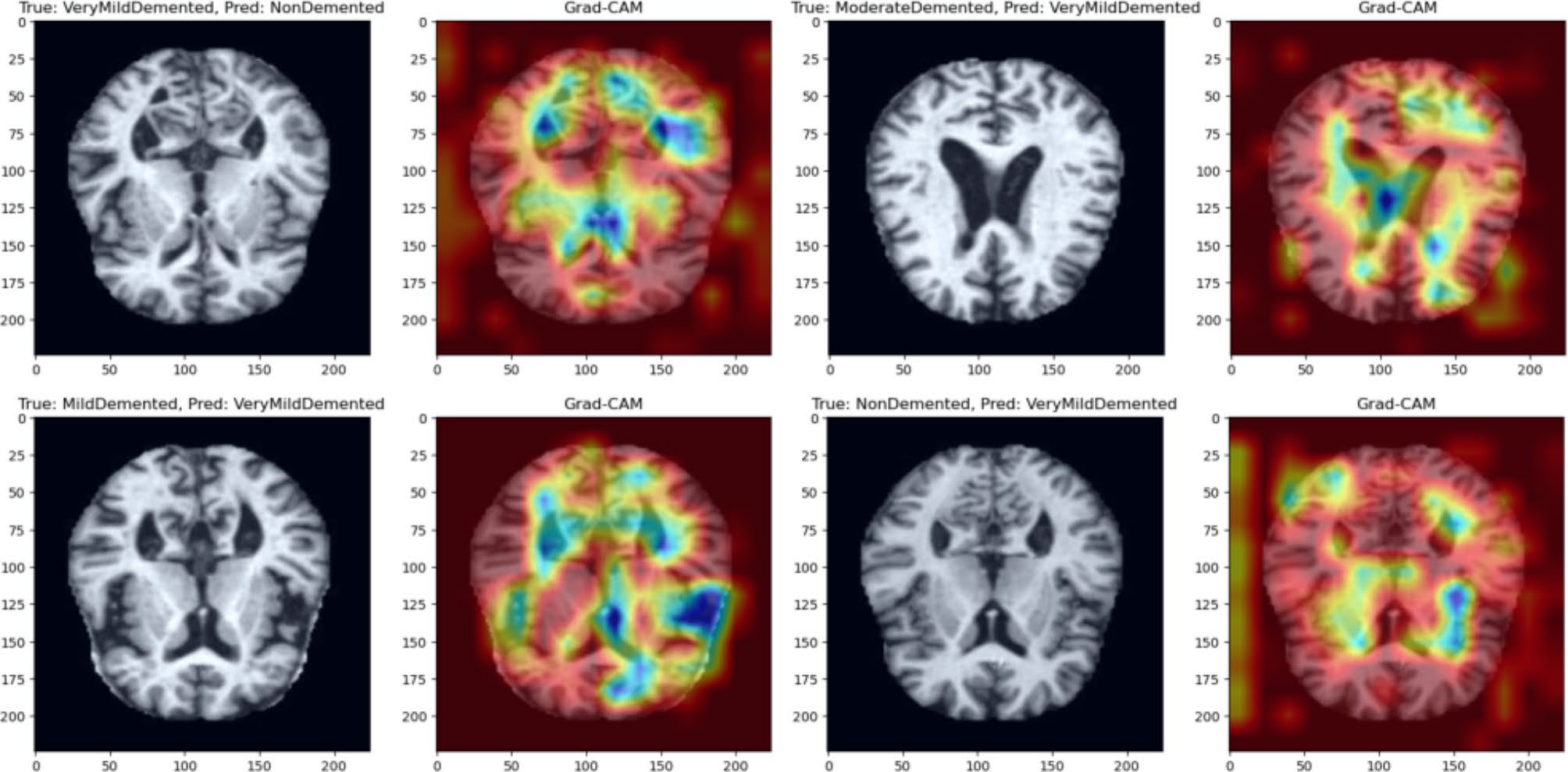
Grad-CAM visualizations of misclassified MRIs.

Figure 3 shows the Grad-CAM Visualizations of Misclassified MRIs. Misclassified MRIs of four Alzheimer’s classes and their corresponding Grad-CAM heatmaps. For each pair, the left image shows the original MR image, while the right image displays the Grad-CAM heatmap. The true class and predicted class for each image are labeled, providing insights into the model’s misclassification patterns. Grad-CAM highlights the regions of the image that the model focused on during its prediction process, with warmer colors (red/yellow) indicating areas that contributed most to the model’s decision, and cooler colors (blue/green) showing less influential regions. These visualizations reveal potential challenges in differentiating between MR images of Alzheimer’s types.

### Experimental setup

This study’s deep neural networks were developed using Matlab version 9.8, 2020a. All experiments were conducted on a machine with an Intel Core (TM) i7-6500 CPU @ 2.50 GHz and 16 GB RAM. In this research, we applied CNN pre-trained neural networks through transfer learning to classify AD. This research utilizes pre-trained models such as AlexNet, GoogleNet, and MobileNetV2 for classification purposes.

We carefully examined how changing different parameters could help us achieve the best accuracy for our model. We experimented with various epochs and different initial learning rates during training to understand their impact on performance. After that, we investigated which solver would work best with the parameters we tested. To do this, we tried out several solvers with three different architectures. Our goal was to fully fine-tune the model, ensuring it performs optimally based on our findings.

**Table 4:** Detail of parameters for different architecture.

Architecture	Implemented solvers	Initial learning rate	No. of epochs
AlexNet	SGDM, ADAM, RMSPROP	0.001	25
GoogleNet	SGDM, ADAM, RMSPROP		
MobileNetV2	SGDM, ADAM, RMSPROP		

Table 4 shows the architectures used in the study, the solvers applied, initial learning rates, and the number of training epochs. AlexNet, GoogleNet, and MobileNetV2 all used SGDM, ADAM, and RMSPROP as solvers, with a default batch size of 128. The initial learning rate is set to 0.001, and each model was trained for 25 epochs.

Since these pre-trained models have already undergone training on extensive databases of images, they require significantly less time and computing resources for further training than developing new models^45^. These networks are comprised of thousands of neurons and have millions of parameters. AlexNet, with 62.3 million parameters, pioneered deep learning with its three fully connected and five convolutional layers, while GoogleNet’s 22 layers, expanded to 27 with pooling layers, revolutionized neural network architectures. MobileNetV2, with fifty-three deep layers, excels in classifying 100 object categories, leveraging millions of images for training. Although the number of layers in each model differs, some common layers are found in almost all CNN models, including input, convolution, pooling, fully connected, and output layers^42^.

The solver coordinates the network’s forward inference and backward gradients to create parameter updates that aim to reduce the loss. This process is known as model optimization. The solver is in charge of managing the optimization and producing parameter updates. In our study, we used three different solvers to optimize training architectures, as follows^43^:

#### Stochastic gradient descent with momentum (SGDM)

Stochastic gradient descent with momentum (SGDM) is an optimization algorithm that enhances the basic gradient descent approach by introducing a momentum factor. SGDM updates the network’s weights and biases to reduce the loss function by incrementally moving toward the negative gradient of the loss function with each iteration. This momentum factor helps reduce oscillation during parameter updates, leading to smoother convergence towards the optimal solution^43^.

1 $$\delta \ell +1\,=\,\delta \ell - a\nabla E(\delta \ell ){\text{ }}+g(\delta \ell - \delta \ell - 1)$$

Where ℓ represents the number of iterations, α > 0 specifies the learning rate, δ represents the parameter vector, E(δ) denotes the loss function, and γ indicates the contribution of the previous gradient step to the current iteration.

#### Root mean square propagation (RMSPROP)

RMSPROP is one method that stores a moving average using the squares of the parameter gradients by element^43^.

2 $$v\ell ={\rm B}2v\ell - 1+(1 - {\rm B}2){\text{ }}[\nabla E(\delta \ell )]2$$

vℓ: Exponentially Weighted Moving Average (EWMA) of the square of gradients at layer ℓ. B2: Hyperparameter scaling the previous EWMA term. (1−B2): Complement of B2, controlling direct contribution of current gradient’s square. [∇E(δℓ)]^2: Square of the gradient of the loss function. Multiplying B2 with vℓ scales the previous EWMA term. Multiplying (1−B2) by [∇E(δℓ)]^2 scales the current gradient’s square. Combined, they update parameters during optimization, often used in algorithms like Adam for stochastic optimization. Where B2 reflects the moving average’s decay rate. The squared gradient’s average length is equal to 1/ (1−B2), with the value of 10, 100, and 1000 parameter updates, respectively^43^.

3 $$\delta \ell +1\,=\,\delta \ell - a\nabla E(\delta \ell ){\text{ }}/\surd v\ell + \in$$

Here, each element is divided separately When using RMSProp, the learning rates for parameters with small gradients are effectively increased, while the learning rates for parameters with high gradients are effectively decreased. To prevent division by zero, a small constant ɛ is included.

#### Adaptive moment estimation (ADAM)

ADAM employs a parameter similar to RMSPROP with the addition of a momentum term. It maintains track of the parameter gradients and their squared values using a moving average based on elements^43^.

4 $$m\ell = {\rm B}1m\ell - 1 + (1 - {\rm B}1)\nabla E{\text{ }}(\delta \ell )$$

5 $$v\ell =B2v\ell - 1+(1 - B2){\text{ }}[\nabla E(\delta \ell )]2$$

Where the decay rates for the training choices squared gradient decay factor and gradient decay factor are B1 and B2, respectively. To update the network settings, Adam employs moving averages as^43^:

6 $$\delta \ell +1\,=\,\delta \ell - aml/\surd vl+\smallint$$

Where ɛ with the help of the epsilon training option. If gradients across numerous iterations are the same, using a moving average of the gradient enables parameter changes to build momentum in one direction. When noise dominates the gradients, the gradient’s moving average becomes smaller, resulting in smaller parameter updates.

**Fig. 4:**
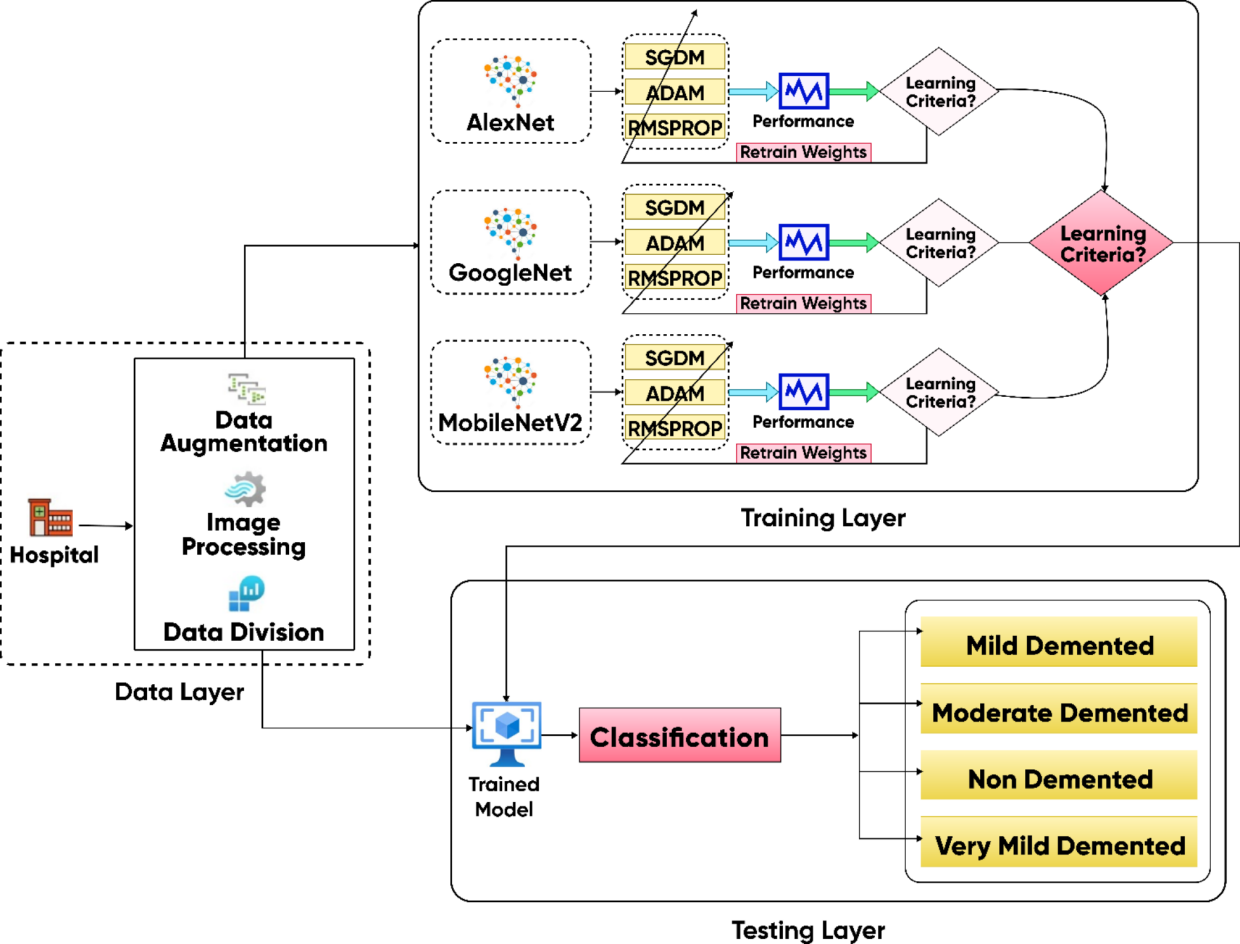
Proposed model for detection of AD from MRI images.

The proposed model is shown in Fig. 4. The diagram represents a comprehensive workflow for proposed model, divided into three key layers: data layer, training layer, and testing layer. Detailed explanation of each layer is given as:

##### Data layer

Data Source: Data is collected from hospitals, consisting of Alzheimer’s patient scans.

Data Augmentation & Image Processing: To improve the robustness of the model, data augmentation techniques were implemented such as flipping, rotation, scaling, etc. This step helps in creating more diverse training data and prevents overfitting. Image processing techniques also include that the images are converted into a suitable format for the model.

Data Division: After preprocessing, the dataset is divided into training, and testing sets to ensure proper evaluation of the model’s performance.

##### Training layer

Model selection: Three different pre-trained convolutional neural networks (CNNs) are utilized: AlexNet, GoogleNet, and MobileNetV2. These models are known for their capability to extract significant image features.

Optimizer choice: Three different optimization techniques like SGDM, ADAM, and RMSPROP are used for each architecture. These techniques adjust the model’s weights during training to make the model perform better.

Learning criteria: The process involves a decision block to determine whether the model has met the predefined performance criteria. If the model does not meet the expected accuracy or other criteria, it loops back for weight retraining, optimizing until satisfactory results are achieved.

##### Testing layer

The testing phase involves using unseen images to evaluate the model’s generalization capability.

Classification: The trained model classifies the input images into one of the four categories: mild demented, moderate demented, very mild demented, and non-demented. This classification assists in detecting the level of Alzheimer’s disease in the patient based on the provided medical images.

The evaluation matrices listed below were employed to assess the performance of the implemented models^7^:

7 $$Accuracy~=\frac{{\varepsilon + \propto }}{{\varepsilon +\gamma + \propto +\delta }}$$

8 $$Specificity~=\frac{ \propto }{{ \propto +\gamma }}$$

9 $$\operatorname{Re} call~or~Sensitivity=\frac{\varepsilon }{{\varepsilon +\delta }}$$

10 $$\Pr ecision~=\frac{\varepsilon }{{\varepsilon +\gamma }}$$

11 $$F1~score~=\frac{{2*~\Pr ecision~*~\operatorname{Re} call~}}{{~\Pr ecision~+~\operatorname{Re} call~}}$$

12 $$Misclassification~rate=100~ - ~(\frac{{\varepsilon + \propto }}{{\varepsilon +\gamma + \propto +\delta }})$$

Where ε represents the predicted true positive value, ∝ represents the predicted true negative value, γ represents the predicted false positive value, and δ represents the predicted false negative value.

## Results

For classification purposes, we divided the images into four classes according to the CDR scores: CDR-0 (Non-Demented), CDR-0.5 (Very Mild-Demented), CDR-1 (Mild-Demented), and CDR-2 (Moderate-Demented). We implemented three distinct CNN models (AlexNet, GoogleNet, and MobileNetV2), testing each model with three different solvers (SGDM, ADAM, RMSPROP) to identify which model performs best with each solver. In this section, we provided detailed results for each model alongside the solver it performed best with, ensuring that the results section is brief. Table 5 illustrates the number of epochs, initial learning rate, number of iterations, implemented solvers, and accuracy performance of each model.

**Table 5:** Experimental results of pre-trained models.

Model name	No. of epochs	Initial learning rate	No. of iterations	Implemented solvers	Accuracy performance (%)
AlexNet	25	0.001	2000	SGDM	98.4
				ADAM	**99.4**
				RMSPROP	89.6
GoogleNet	25	0.001	2000	SGDM	95.1
				ADAM	**98.0**
				RMSPROP	87.5
MobileNetV2	25	0.001	2000	SGDM	**96.5**
				ADAM	94.1
				RMSPROP	84.5

Significant values are given in bold.

We calculated the true positive, true negative, false positive, and false negative values for each class using the models that yielded the best results with specific solvers. Based on these values, we performed additional calculations to assess the implemented architectures’ class-wise precision, misclassification rate, accuracy, recall, specificity, and F1 score. After selecting the optimal hyper-parameters, we evaluated the performance of these models using all the data from the training and testing subsets

### Class-wise detailed results of implemented architectures trained with optimal solvers

#### Performance evaluation of AlexNet model trained with ADAM solver

To evaluate the effectiveness of different CNN architectures, we tested the AlexNet model using the ADAM solver on a dataset of MRI images. We assessed the performance of the AlexNet model trained with the ADAM solver on four classes of MRI images sourced from the Kaggle dataset. Our findings revealed that the model attained an accuracy of 99.4%.

**Fig. 5:**
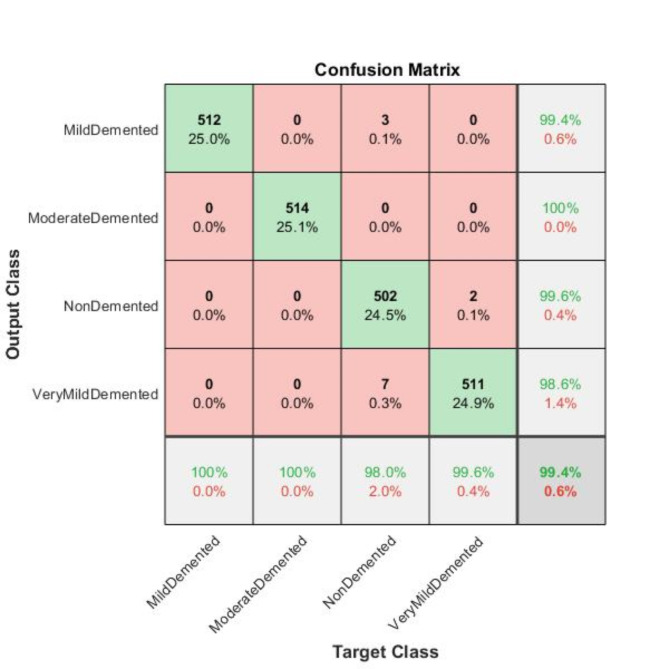
Confusion matrix of AlexNet model trained with the ADAM solver.

**Fig. 6:**
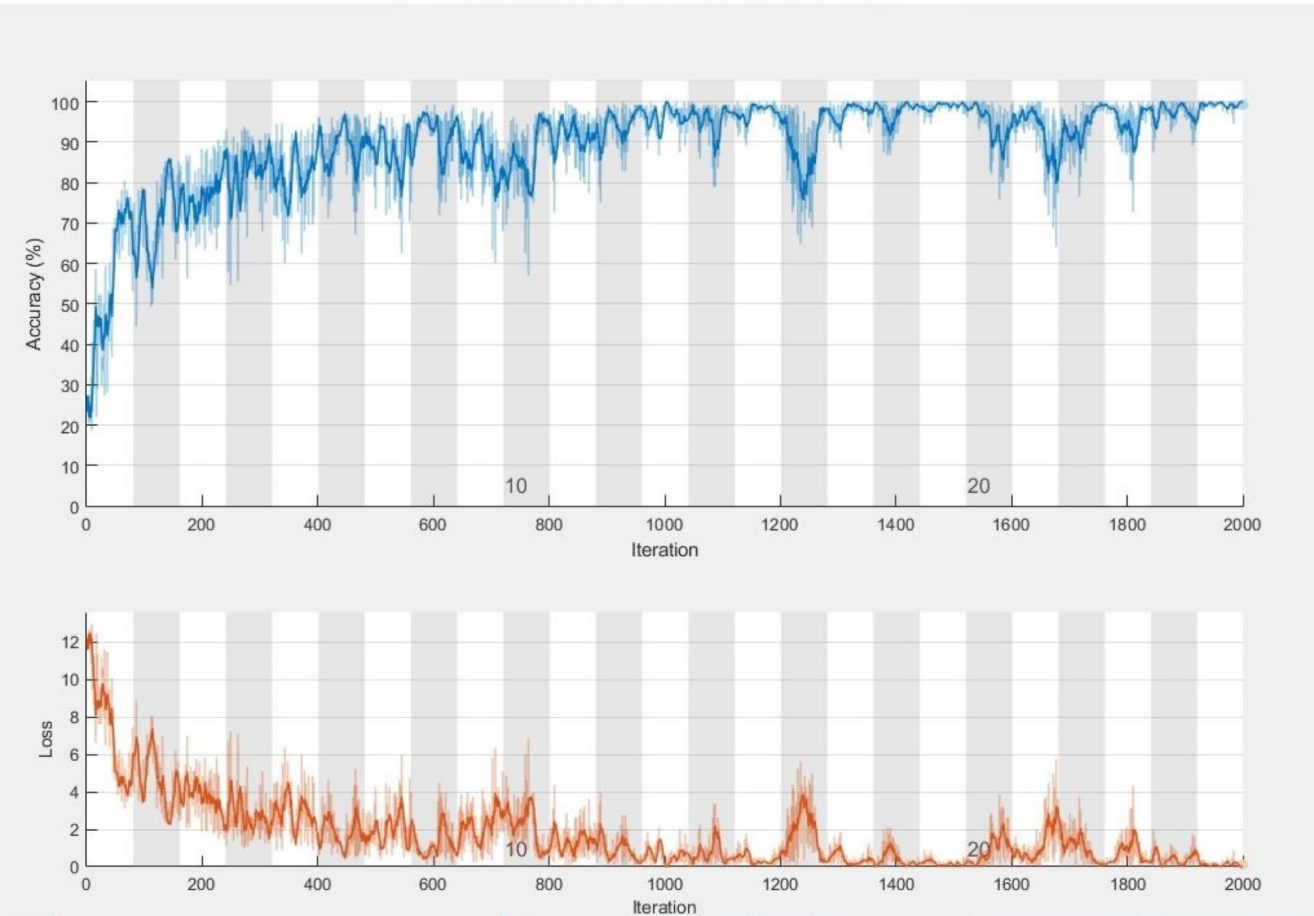
Progress of AlexNet model: accuracy and loss over epochs and iterations.

We evaluated the class-wise precision and recall of the model (Table 6; Figs. 5 and 6). Our findings revealed that the model achieved 99.4% precision and 100% recall for the Mild-Demented class, 100% precision and 100% recall for the Moderate-Demented class, 99.6% precision and 98.0% recall for the Non-Demented class, and 98.6% precision and 99.6% recall for the Very Mild-Demented class. Next, we assessed the number of correctly classified images in each class. Out of the 515 images in the Mild-Demented class, 512 were correctly classified, with three misclassified. All 514 images in the Moderate-Demented class were correctly classified. Of the 504 images in the Non-Demented class, 502 were correctly classified, with two misclassified. Lastly, out of the 518 images in the Very Mild-Demented class, 511 were correctly classified, with seven misclassified.

**Table 6:** Class-wise performance of AlexNet according to Accuracy, Precision, F1 score, recall, specificity, and Misclassification Rate.

Classes	True Positive (TP)	True Negative (TN)	False Positive (FP)	False Negative (FN)	Accuracy	Precision	F-1 Score	Recall	Specificity	Misclassification Rate
Mild Demented	512	1536	3	0	99.85%	99.42%	99.71%	100.00%	99.81%	0.001462701
Moderate Demented	514	1537	0	0	100.00%	100.00%	100.00%	100.00%	100%	0
Non Demented	502	1537	2	10	99.41%	99.60%	98.82%	98.05%	99.87%	0.005850804
Very Mild Demented	511	1531	7	2	99.56%	98.65%	99.13%	99.61%	99.54%	0.004388103

Analyzing the performance of the AlexNet model, which was trained with the ADAM solver, across various stages of Alzheimer’s Disease, namely: mild-demented, moderate-demented, non-demented and very mild-demented disorders has also been done in Table 6. Performance was quantified in terms of Accuracy, Precision, F1-Score, Recall, Specificity and Misclassification Rate, for the respective cases. While the model performed quite well, we noticed very few instances of misclassification especially in the Non Demented and Very Mild Demented levels. The model performed exceptionally well for the Mild-Demented and Moderate-Demented classes, with the Moderate-Demented classification showing 0% misclassification, indicating the accuracy of the model in differentiating moderate dementia from other levels. In contrast, there were very low (0.00585 and 0.00439, respectively) misclassification rates for the Non-Demented and Very Mild-Demented classes, which indicate that it may be somewhat difficult to the subject who can carry the two stages. More specifically in the Non-Demented class, there were 10 False Negatives (FN) and 2 False Positives (FP), which means that the Recall (98.05%) and F1-Score (98.82%) were slightly low than in other classes. This could mean that some Non-Demented individuals have dementia stage features overlapped with them which makes it difficult for the model to distinguish between very early dementia, and completely healthy individuals, as is the case here. Subtle structural changes in the brain that do not manifest as clinical symptoms may contribute to these misclassifications.

There were two False Negatives (FN), and seven False Positives (FP) in the Very Mild-Demented class, which provided a Precision of 98.65 and a Recall of 99.61. The higher rate of False Positives in this class indicates that the model at times, misclassified Non-Demented patients as those with Very Mild-Dementia. This may be attributed to the thin line that exists between normal aging and a very mild form of cognitive impairment that exhibits no structural changes in the brain at this stage — changes that can easily be confused with variations within normal aging patterns. Possible Reasons for misclassifications could be the feature overlap– Non-Demented and Very mild Demented individuals tend to have indistinct MRI characteristics in most cases as in the case of atrophy or thinning of the hippocampus. Entering into some of these changes in the brain at this stage is a feature that may not be easy to appreciate without finer techniques of feature extraction. CNNs are great for facilitating the process of feature extraction. When it comes to MRI scans, there are usually some finer points that unfortunately this model fails to capture most especially for cases that border on the extremes. Transferring learning might be effective but there would be need for more tweaks or more complex architectures to enhance the results.

**Fig. 7:**
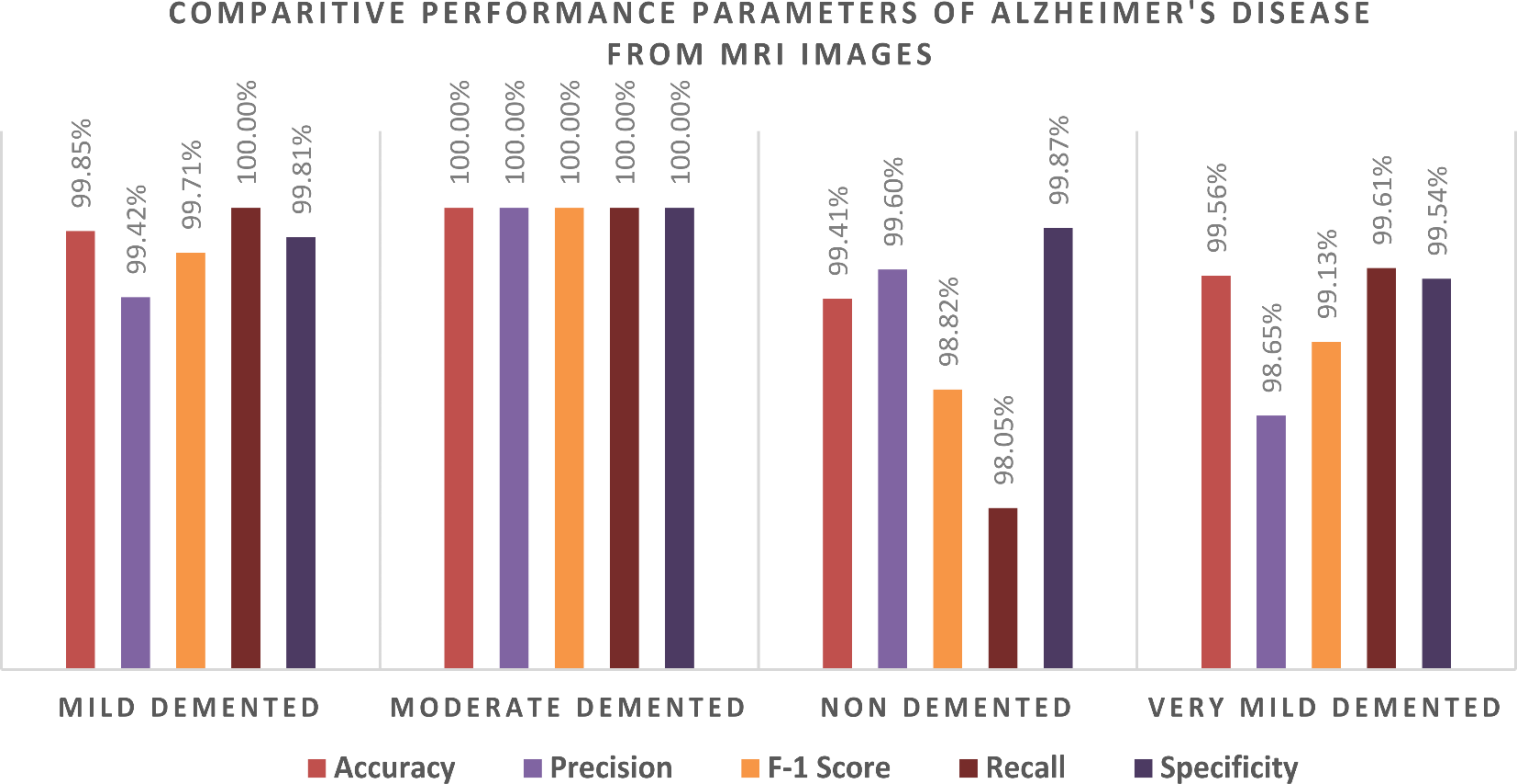
Graphical representation of the class-wise performance of the AlexNet Model according to Accuracy, Precision, F1 Score Recall, and Specificity for each class.

All calculated values described in Table 6 are shown graphically in Fig. 7.

#### Performance evaluation of GoogleNet model trained with ADAM

We assessed the performance of the GoogleNet model trained with the ADAM solver on four classes of MRI images sourced from the Kaggle dataset. Our findings revealed that the model attained an accuracy of 98.0%.

**Fig. 8:**
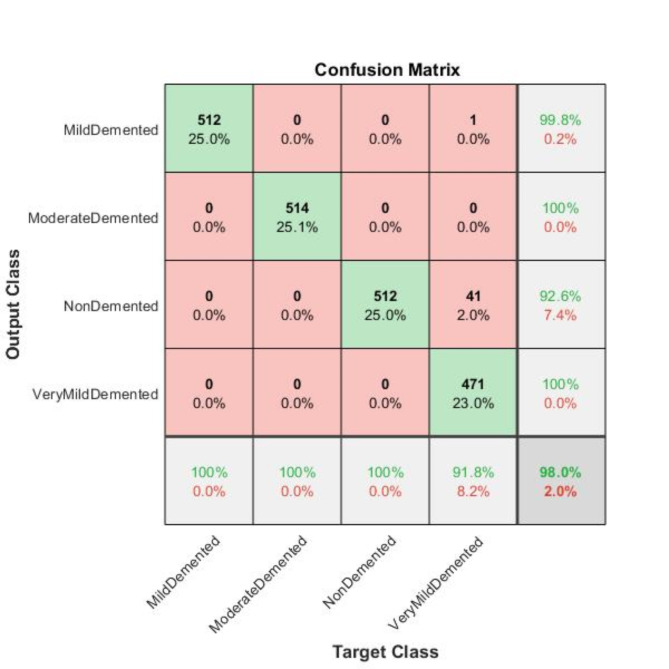
Confusion matrix of GoogleNet model trained with the ADAM solver.

**Fig. 9:**
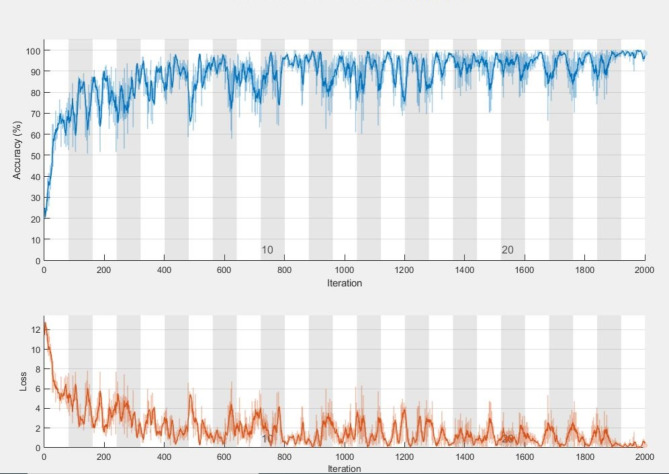
Progress of GoogleNet model: accuracy and loss over epochs and iterations.

We evaluated the class-wise precision and recall of the model (Table 7; Figs. 8 and 9). Figure 9 demonstrates that we attained a precision of 99.8% and a recall of 100% for the Mild-Demented class, 100% precision and recall for the Moderate Demented class, 92.6% precision and 100% recall for the Non-Demented class, and 100% precision and 91.8% recall for the Very Mild Demented class. Of the 513 images in the Mild-Demented class, 512 were correctly classified, with one misclassified. All 514 images in the Moderate-Demented class were correctly classified. Of the 533 images in the Non-Demented class, 512 were correctly classified, with 41 misclassified. Lastly, all 471 images in the Very Mild-Demented class were correctly classified.

**Table 7:** Class-wise performance of the GoogleNet model, including Accuracy, Precision, F1-Score, recall, specificity, and Misclassification Rate.

Classes	TP	TN	FP	FN	Accuracy	Precision	F1- score	Recall	Specificity	Misclassification rate
Mild Demented	512	1538	1	0	99.95%	99.81%	99.90%	100%	99.94%	0.000487567
Moderate Demented	514	1537	0	0	100.00%	100%	100%	100%	100%	0
Non Demented	512	1498	41	0	98.00%	92.59%	96.15%	100%	97.34%	0.019990249
Very Mild Demented	471	1538	0	42	97.95%	100%	95.73%	91.81%	100%	0.020477816

**Fig. 10:**
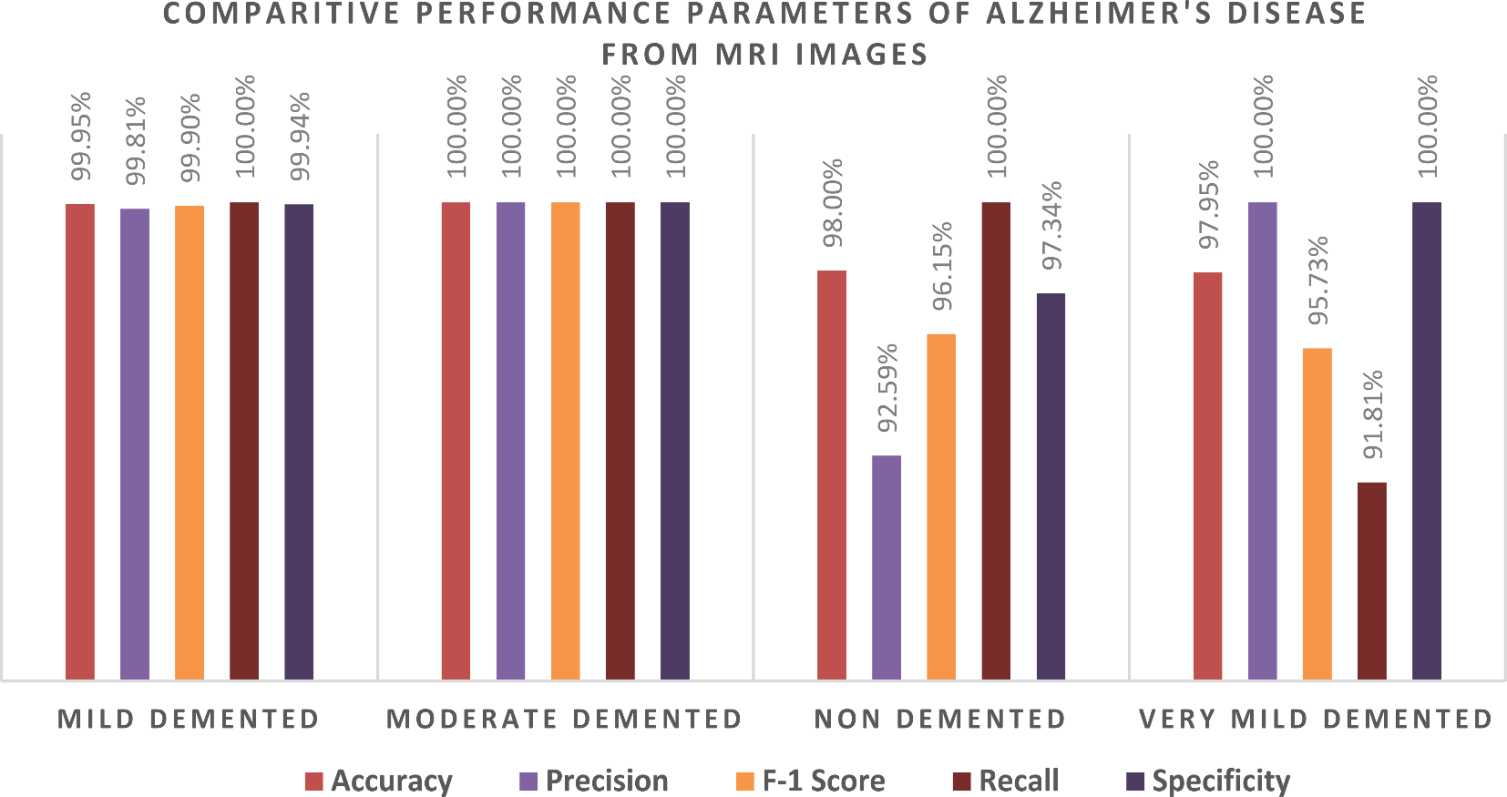
Graphical representation of the class-wise performance of the GoogleNet Model according to accuracy, precision, F1 score recall, and specificity for each class.

Next, we assessed the class-specific GoogleNet trained with ADAM architecture’s performance according to Accuracy, Precision, F-1 Score, Recall, Specificity and Misclassification rate (Table 7; Fig. 10).

For the Mild-Demented class, the following results were attained: Accuracy of 99.95%, F-1 score of 99.90%, Precision of 99.81%, Specificity of 99.94%, Recall of 100%, and a Misclassification rate of 0.000487567. For the Moderate-Demented class, the model achieved an Accuracy, F-1 score, Precision, Specificity, and Recall of 100%, with a 0 Misclassification Rate. For the Non-Demented class, the following results were obtained: Accuracy of 98.0%, F-1 score of 96.15%, Precision of 92.59%, Specificity of 97.34%, Recall of 100%, and a Misclassification rate of 0.019990249. For the Very Mild-Demented class, the results were as follows: Accuracy of 97.95%, F-1 score of 95.73%, Precision of 100%, Specificity of 100%, Recall of 91.81%, and a Misclassification Rate of 0.020477816. The model demonstrated exceptional performance across different dementia classes, achieving high accuracy, precision, recall, and specificity.

Table 7 illustrates the performance results obtained with the GoogleNet model trained using the ADAM solver for a four-stage AD classification namely Mildly-Demented, Moderately-Demented, Non-Demented and Very Mildly-Demented too. Although the model performed well in accuracy, precision, recall and specificity on most of the classes, there are some crucial points of the class misclassification rates, especially in Non-Demented and Very Mild-Demented classes, that should be addressed. As for the Mild-Demented and Moderate-Demented classes, the model performed almost perfectly, achieving a recall and precision of 1, which implies that the model can easily differentiate the above stages from the rest. The misclassification rates are close to zero, The Non-Demented category, on the other hand, had 41 False Positives, which affected the precision to 92.59%, which means that some Non-Demented persons were labeled as demented. Such a misdiagnosis can have implications in healthcare as it can lead to needless interventions or treatments for patients who are wrongly diagnosed with dementia. Even though there is no dementia case that is in this class that was left out, as indicated by the False Negative rate of 0%, the alarming False Positive rate (41 images) compromises the precision increasing chances of overdiagnosis. In a real-life situation, this introduces frustration to both the patients and their relatives as well as unnecessary follow-ups or treatments.

The class of very mild-demented had 42 cases (False Negatives) which were wrongly identified bringing the recall at 91.81%. Recall here suggests that the model failed to determine 42 cases of very mild dementia. When it comes to the precision, it stands at 100%, that is, No Non-Demented patient was misclassified as very mild-demented. However, the low recall shows that the model was able to identify almost 8% of patients who were very mild-demented. For example, this type of underdiagnosis can postpone treatment, therefore the patients may not benefit from early treatment which is fundamental to curbing the advancement of AD. The Very Mild-Demented class presents a paradox; this is an instance where the model manages to detect cases but with some limitations. High precision means that when the model classifies an instance as a case of very mild dementia, it is accurate 100% of the time. Nonetheless, the recall is relatively low (91.81%) and this indicates that there are many actual cases that go undetected which is quite worrisome in a clinical setting where one aims to detect the onset of the illness as early as possible i.e. AD. The False Negatives within this level illustrate losses in regard to identifying the disease at an appropriate stage, hence, allowing room for treatments which may not be helpful to the patient.

#### Performance evaluation of MobileNetV2 model trained with SGDM solver

We assessed the performance of the MobileNetV2 model trained with the SGDM solver on four classes of MRI images sourced from the Kaggle dataset. Our findings revealed that the model attained an accuracy of 96.5%.

**Fig. 11:**
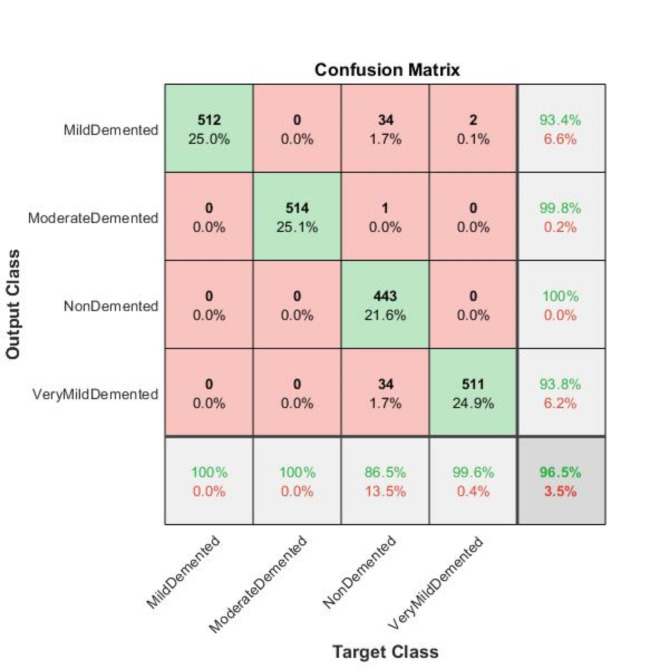
Confusion matrix of MobilenetV2 model trained with the SGDM solver.

**Fig. 12:**
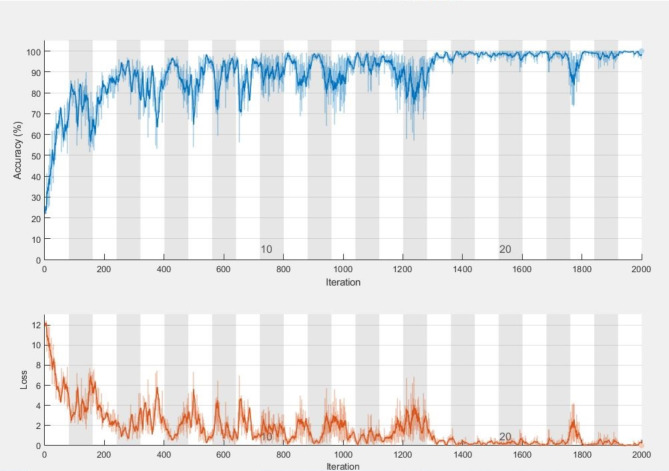
Progress of MobileNetV2 model: accuracy and loss over epochs and iterations.

We evaluated the class-wise precision and recall of the model (Table 8; Fig. 11, and Fig. 12). Figure 11 illustrates that we achieved 93.43% precision and 100% recall for the mild-demented class, 99.8% precision and 100% recall for the moderate-demented class, 100% precision and 86.5% recall for the non-demented class, and 93.8% precision and 99.6% recall for the very mild-demented class. Of the 548 images in the mild-demented class, 512 were correctly classified, with 36 misclassified. Of the 515 images in the moderate-demented class, 514 were correctly classified, with one misclassified. All 443 images in the very non-demented class were correctly classified. Lastly, out of the 545 images in the very mild-demented class, 511 were correctly classified, with 34 misclassified.

**Fig. 13:**
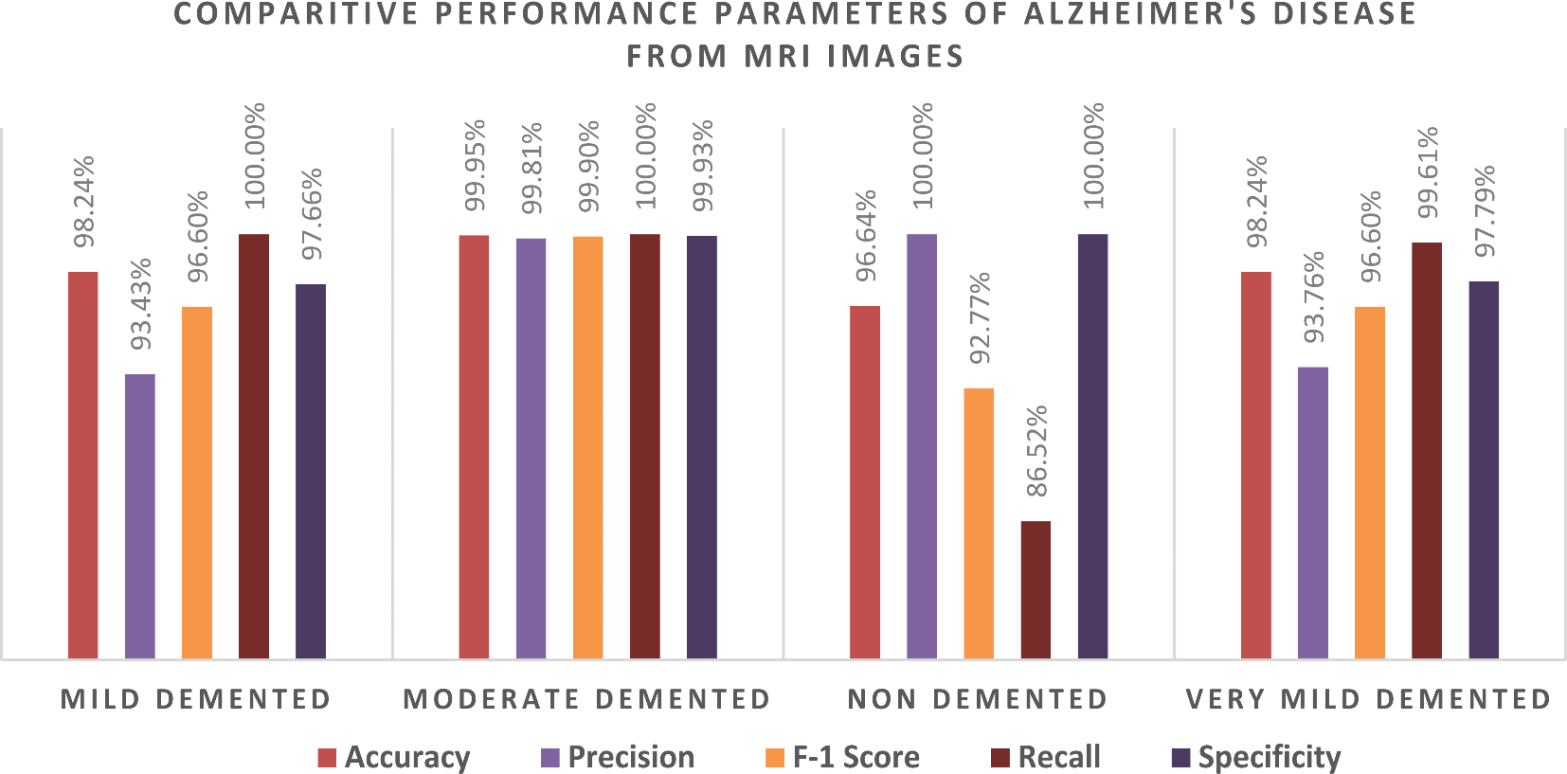
Graphical representation of the class-wise performance of the MobilenetV2 model according to accuracy, precision, F1 score recall, and specificity for each class.

Next, we assessed the class-specific MobileNetV2 trained with SGDM architecture’s performance according to Accuracy, Precision, F1-Score, Recall, Specificity, and Misclassification rate (Table 8; Fig. 13).

For the Mild-Demented class, we achieved 98.24% Accuracy, 93.43% Precision, 96.60% F1 score, 100% Recall, 97.66% Specificity, and a Misclassification rate of 0.017552413. For the Moderate-Demented class, we achieved 99.95% Accuracy, 99.81% Precision, 99.90% F1 score, 100% Recall, 99.93% Specificity, and a Misclassification rate of 0.000487567. For the Non-Demented class, we attained an Accuracy of 96.64%, a Specificity of 100%, an F1 score of 92.77%, a Recall of 86.52%, a Precision of 100%, and a Misclassification rate of 0.033642126. Moreover, for the Very Mild-Demented class, we achieved 98.24% Accuracy, 93.76% Precision, 96.60% F1 score, 99.61% Recall, 97.79% Specificity, and a Misclassification rate of 0.017552413.

**Table 8:** Class-wise performance of the MobilenetV2 model, including accuracy, precision, F1-score, recall, specificity, and misclassification rate.

Classes	TP	TN	FP	FN	Accuracy	Precision	F1- Score	Recall	Specificity	Misclassification Rate
Mild Demented	512	1503	36	0	98.24%	93.43%	96.60%	100%	97.66%	0.017552413
Moderate Demented	514	1536	1	0	99.95%	99.81%	99.90%	100%	99.93%	0.000487567
Non Demented	443	1539	0	69	96.64%	100%	92.77%	86.52%	100%	0.033642126
Very Mild Demented	511	1504	34	2	98.24%	93.76%	96.60%	99.61%	97.79%	0.017552413

Table 8 shows how the MobileNetV2 model was evaluated in four stages of dementia: mild-demented, moderate-demented, non-demented, and very mild-demented. The system performs well at 99.66% and 97.31% accuracy rates in the moderate-demented and non-demented classes respectively. However, several errors of classification are present in the mild-demented and the very mild-demented classes which need to be evaluated, understood, and resolved.

When it comes to the mild-demented class, it shows a 1.76% misclassification rate with 36 false positives (FP) leading to a Precision of 93.43% and an F1-Score of 96.60%. Likewise, when it comes to the Very Mild-Demented class, it also demonstrates a 1.76% misclassification rate with 34 False Positives (FP) and 2 False Negatives (FN) giving rise to 93.76% precision and an F1-Score of 96.60%. The aforementioned misclassifications indicate that the model experiences difficulty differentiating between the early stages of dementia, such as Mild and Very Mild Demented, and other stages. This may be due to the absence of distinct brain changes since these changes start in milder forms of dementia which may also be present in the elderly or in the more advanced forms of dementia. Characteristics observed via MRI may include atrophy of the hippocampus or thinning of cortical levels which may not be visibly pronounced; causing the model to confuse Mild-Demented and Very Mild-Demented patients with those who do not have dementia but are in a more advanced stage.

In the classification of dementia, the problem of class overlaps in the early-stage diagnosis is a serious one, as it is often the case that changes in the brain are subtle or non-specific. Here, the term False Positives point to the tendency of the model to incorrectly label Non-Demented subjects as Mild-Demented and Very Mild-Demented individuals. In practice, this might lead to an increase in the errors of diagnosis among the population, including the treatment of patients with normal cognition. The model performs exceptionally well in the Moderate-Demented class recording an Accuracy of 99.95%, Precision of 99.81%, and a mere 0.05% misclassification rate. This implies that the model is quite effective in classifying more advanced forms of dementia as there are gross changes in the brain that can be easily identified. In contrast, the Non-Demented class shows a rate of 3.36% of misclassification with 69 False Negatives (FN) recorded which resulted in Recall of 86.52% and F1-Score of 92.77% respectively. The False Negatives in this class may suggest that some Non-Demented individuals are being.

Using the precision and recall metrics it is possible to draw conclusions about the clinical relevance of the model. For example, high precision means that when the model classifies a case as positive Mild-Demented or Very Mild-Demented, this is most likely to be correct, which is important in avoiding becoming overzealous in pursuit of a diagnosis where one does not exist that may lead to harmful treatments. The non-demented and mildly demented categories being associated with lower recall means that, in fact, the model may be misclassifying some early-stage patients, which would lead to some cases being undiagnosed. In real-world clinical practice, it becomes imperative for a physician to be able to detect dementia among patients since it is known that early intervention would mitigate the course of the disease. The model exhibits low recall in the Non-Demented and Very Mild-Demented classes, which implies that some cases of early-stage dementia in practice may go undetected, leading to a postponement in treatment. This is particularly true here where improving recall in these classes is very important to maximize the detection of cognitive impairment at its most early stages for timely intervention.

### Class-wise performance summary for all architectures

As shown in Table 9, the AlexNet, GoogleNet, and MobileNetV2 models were evaluated across four classes: mild demented, moderate demented, non demented, and very mild demented. For Mild Demented, all models had good results, and all the models were examined to have perfect recall, and minimized misclassification were noticed but precision of MobileNetV2 was not much better, which was 93.43%. In Moderate Demented, both AlexNet and GoogleNet performed perfectly across all metrics (100%), and MobileNetV2 also showed strong performance but with a slight misclassification rate. In non Demented, overall results are encouraging but recall results of MobileNetV2 and Precision of GoogleNet are slightly low. In Very Mild Dementia, it was found that GoogleNet had perfect precision but low recall (91.81%), while AlexNet had excellent precision (98.65%) and high recall (99.61%).

**Table 9:** Summary of class-wise performance for AlexNet, GoogleNet, and MobileNetV2 models based on recall, precision, and misclassification rate.

Classes	Model	Recall	Precision	Misclassification rate
Mild demented	AlexNet	100.00%	99.42%	0.00146
	GoogleNet	100.00%	99.81%	0.00049
	MobileNetV2	100.00%	93.43%	0.01755
Moderate demented	AlexNet	100.00%	100.00%	0
	GoogleNet	100.00%	100.00%	0
	MobileNetV2	100.00%	99.81%	0.00049
Non demented	AlexNet	98.05%	99.60%	0.00585
	GoogleNet	100.00%	92.59%	0.01999
	MobileNetV2	86.52%	100.00%	0.03364
Very mild demented	AlexNet	99.61%	98.65%	0.00439
	GoogleNet	91.81%	100.00%	0.02048
	MobileNetV2	99.61%	93.76%	0.01755

### External evaluation

#### Performance of the proposed model on the OASIS dataset

Our experiments revealed that the AlexNet model trained with the ADAM solver achieved the highest performance. To validate this finding, we conducted an external evaluation to assess the performance of this model using four classes of MRI images from the OASIS dataset. We found that the model achieved 98.2% classification accuracy on the OASIS dataset using the AlexNet architecture and ADAM solver.

**Fig. 14:**
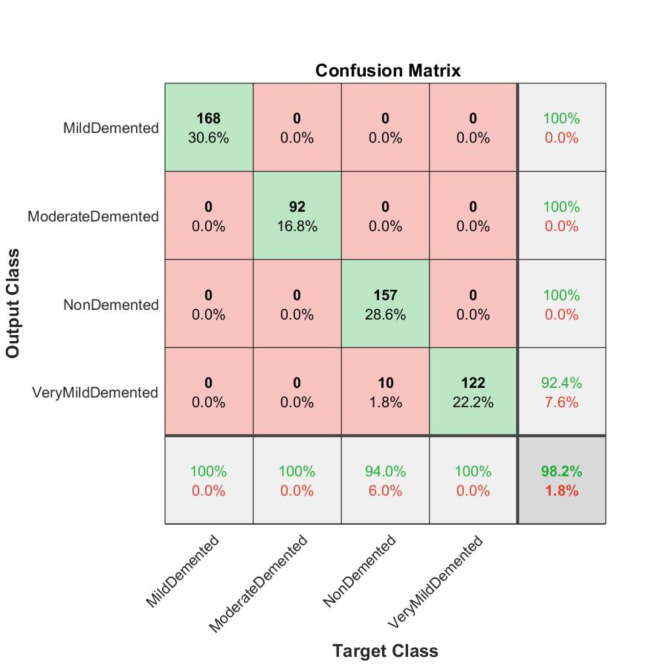
Confusion matrix of the employed model on the OASIS dataset.

**Fig. 15:**
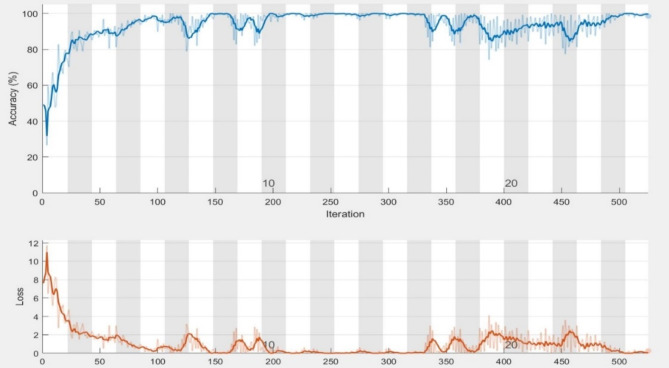
Training progress of the proposed model on the OASIS dataset.

We evaluated the model’s class-wise precision and recall (Figs. 14 and 15). Our findings revealed that the model achieved 100% precision and recall for the mild-demented class and 100% precision and recall for the moderate-demented class. Additionally, it achieved 100% precision and 94.0% recall for the Non-Demented class and 92.4% precision and 100% recall for the very mild-demented class. We evaluated the class-specific proposed model’s performance according to accuracy, precision, F1- score, recall, specificity, and misclassification rate (Table 10; Fig. 11).

For the mild-demented class, we achieved 100.00% Accuracy, 100.00% Precision, 100.00% F1-Score, 100.00% Recall, 100.00% Specificity, and a Misclassification Rate of 0. For the Moderate-Demented class, we achieved 100.00% Accuracy, 100.00% Precision, 100.00% F1-Score, 100.00% Recall, 100.00% Specificity, and a Misclassification Rate of 0. For the Non-Demented class, we achieved 98.18% Accuracy, 100.00% Precision, 96.91% F1 Score, 94.01% Recall, 100.00% Specificity, and a Misclassification Rate of 0.018214936. For the Very Mild-Demented class, we achieved 98.24% Accuracy, 92.42% Precision, 96.06% F1-Score, 100.00% Recall, 97.76% Specificity, and a Misclassification Rate of 0.017574692.

The class-specific proposed model demonstrated remarkable performance across various dementia classes, achieving high accuracy, precision, recall, and specificity.

**Table 10:** Class-wise performance of the proposed model, including accuracy, precision, F1-score, recall, specificity, and misclassification rate.

Classes	TP	TN	FP	FN	Accuracy	Precision	F1- Score	Recall	Specificity	Misclassification rate
Mild demented	168	371	0	0	100.00%	100.00%	100.00%	100.00%	100.00%	0
Moderate demented	92	457	0	0	100.00%	100.00%	100.00%	100.00%	100.00%	0
Non demented	157	382	0	10	98.18%	100.00%	96.91%	94.01%	100.00%	0.018214936
Very Mild demented	122	437	10	0	98.24%	92.42%	96.06%	100.00%	97.76%	0.017574692

**Fig. 16:**
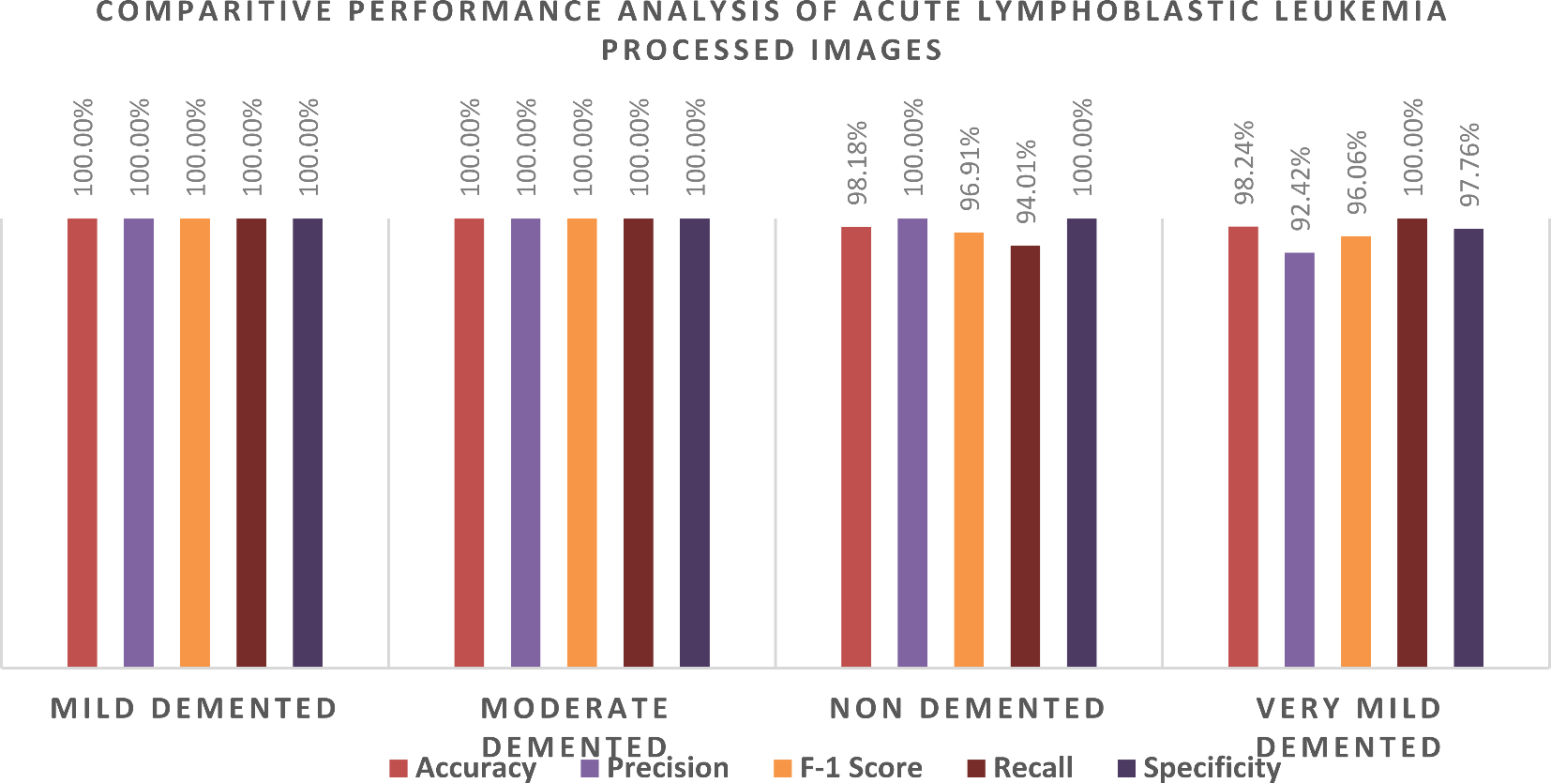
Graphical representation of the class-wise performance of the Proposed Model according to Accuracy, Precision, F1 Score Recall, and Specificity for each class.

In Fig. 16, each bar reflects the corresponding metric for each class, with 100.00% values for most metrics in mild demented and moderate demented classes, and slightly lower values for non-demented and very mild demented. Specifically, non-demented has a Recall of 94.01% and F1 Score of 96.91%, while Very Mild Demented shows a Precision of 92.42% and F1 Score of 96.06%.

#### Performance comparison of proposed model on multiple datasets

In this research paper, we proposed a novel approach for early detection of Alzheimer’s disease using MRI scans and transfer learning techniques with pre-trained CNNs. Our study focused on three distinct CNN architectures, AlexNet, GoogleNet, and MobileNetV2, trained using three different solvers: SGDM, ADAM, and RMSPROP. We tested the models on two datasets: the Kaggle dataset and the OASIS dataset. The choice of solver significantly influenced the performance of the models, as the ADAM solver consistently yielded the highest accuracy across all architectures. SGDM and RMSPROP also produced competitive results but generally lagged behind ADAM.

The AlexNet model achieved the highest accuracy of 99.4% on the Kaggle dataset when trained with the ADAM solver as shown in Table 10, followed by the GoogleNet model with an accuracy of 98.2% and the MobileNetV2 model with an accuracy of 96.5%. The class-wise evaluation of the models revealed their ability to classify different stages of Alzheimer’s disease accurately.

In our research, we identified several constraints, including the dataset size, and the model’s generalizability to different datasets. To enhance our evaluation, we utilized an external dataset called OASIS, which allowed us to assess how well our proposed model performs on unknown MRI images. The results from this new dataset were encouraging. We compared our findings with the previous studies, which utilized the same datasets from Kaggle and OASIS as shown in Tables 11 and 12. Our results were indeed encouraging. The results align closely with the expected outcomes, showcasing the model’s effectiveness. The performance can be significantly enhanced by meticulously fine-tuning the model, adjusting parameters such as the number of epochs and initial learning rate, and, most importantly, identifying the optimal solver.

In the context of Alzheimer’s disease detection, the ADAM optimizer stands out due to its ability to adaptively adjust the learning rate for each parameter. This feature enhances the model’s ability to learn from complex data, such as MRI images, by efficiently handling sparse gradients that often occur in medical imaging tasks.

The proposed model is designed to assist doctors in automatic disease detection. These findings can enhance clinical Alzheimer’s disease detection by providing more accurate and reliable models for identifying the disease in patients. Improved detection methods can lead to earlier diagnosis and better treatment strategies.

**Table 11:** Comparison of AD classification results with existing studies on the Kaggle dataset.

	Year	Data	Model/Classifier	Accuracy
Mggdadi et al. ^45^	2021	Kaggle Dataset	CNN (VGG 16)	70.30%
Suganthe et al. ^46^	2021	Kaggle Dataset	Deep CNN	79.12%
Murugan et al. ^47^	2021	Kaggle Dataset	CNN	95.30%
Yildirim et al. ^49^	2020	Kaggle Dataset	CNN(Resnet50,Densenet201)	96.6%
Proposed model	**2024**	**Kaggle dataset**	**CNN**	**99.4%**

Significant values are given in bold.

**Table 12:** Comparison of AD classification results with existing studies on OASIS dataset.

	Year	Data	Model/Classifier	Accuracy
Battineni et al. ^48^	2021	OASIS Dataset	CNN	83.3%
Islam et al. ^33^	2017	OASIS Dataset	CNN (Inception Network)	73.75%
Sethi et al. ^44^	2022	OASIS Dataset	CNN, SVM	86.2%
Proposed model	**2024**	**OASIS Dataset**	**CNN**	**98.2%**

Significant values are given in bold.

## Limitations

While our proposed approach for Alzheimer’s disease detection using transfer learning and pre-trained CNN models has shown promising results, several limitations must be acknowledged. Our models rely on pre-trained networks, which were initially trained on large-scale datasets unrelated to medical images. This may result in a suboptimal feature extraction for MRI data compared to training CNNs from scratch on medical datasets, although the latter would require much larger labeled datasets. Although the model performed well on the Kaggle and OASIS datasets, the generalizability of the model to other MRI datasets remains untested. MRI data from different sources might vary due to differences in imaging protocols, patient demographics, and scanner settings, which could affect model performance. Despite using transfer learning to reduce the computational cost, the fine-tuning process still requires significant computational power and time for training, especially when testing different solvers and models. Although the OASIS dataset is a useful tool for detecting Alzheimer’s disease, it contains built-in restrictions that could affect how broadly applicable, our findings are:

1. Imaging techniques: The standard techniques used to capture images in this database may differ from those used in other imaging datasets or in clinical practice. When evaluated on fresh and varied datasets, models may perform differently due to variations in the SUBJECTS involved, such as scanners, resolution, and imaging sequences.
2. Demographic variability: When using a resource like OASIS, researchers are limited to using data taken from a group of people who fall into particular age and geographic categories. This could introduce bias into the model, making it potentially inaccurate.

To address these limitations and improve the robustness of the model, we have taken the following steps:

3. 1. Use of multiple datasets: yo provide preliminary external validation, we tested the model’s performance across various imaging methods and demographics using two datasets (Kaggle and OASIS).
4. 2. Transfer learning with pretrained models: to take use of a variety of features and optimization strategies, we used transfer learning with three pretrained models (AlexNet, GoogleNet, MobileNetv2) and solvers (ADAM, SGDM, and RMSprop).

## Advantages

Our research provides several key advantages that distinguish it from previous work in Alzheimer’s disease detection. Our use of transfer learning with fine-tuned CNN architectures resulted in a high classification accuracy, with AlexNet achieving 99.4% on the Kaggle dataset, outperforming several state-of-the-art models. By utilizing pre-trained models, we significantly reduced the need for extensive computational resources and time, compared to training models from scratch. This method is quicker to put into action and is more geared towards researchers without any available resources. We compared the effectiveness of different solvers (ADAM, SGDM, RMSprop) with three different CNN architectures and showed how solver selection helps in improving the performance of the model. This method could be modified to suit other medical image classification tasks apart from the detection of Alzheimer’s disease, hence making it quite useful in medical diagnostics.

## Broader impact statement

The proposed model has significant implications for real-world clinical applications, particularly in under-resourced settings:

1. Adoption in resource-limited environments: the suggested model’s computational simplicity, which is attained by combining transfer learning with lightweight architectures like MobileNetv2, makes it ideal for deployment in environments with constrained computational resources. Utilizing pretrained models reduces the requirement for intensive local training, which further reduces computational expenses.
2. Scalability and accessibility: the model can be used in a variety of clinical settings, including ones with varying imaging methods and demographic characteristics, as evidenced by its flexibility with regard to different datasets. This scalability guarantees that a variety of healthcare facilities can use the system.

3. Reducing barriers to adoption: the model’s open-source framework and low computational needs enable its adoption in resource-constrained environments, allowing healthcare providers to implement it without major infrastructure upgrades.

4. Future directions for impact: in order to facilitate clinical acceptance, future research endeavors may concentrate on developing intuitive user interfaces and incorporating the model into current diagnostic procedures. Furthermore, working together with physicians in contexts with limited resources may yield insightful input that helps improve the system for real-world use.

These considerations highlight the potential of the model to make a meaningful impact in real-world healthcare.

## Future work

The work presented here may be developed in several ways in order to make it more solid and applicable to other patient groups. Future studies should proceed to the verification of the models proposed on other MRI datasets from other centers and countries in order to evaluate the efficacy and applicability of the model in various imaging settings. In addition to MRI, imaging modalities such as PET or CT can be combined in a muli-modal deep learning framework which will help increase the sensitivity of diagnosing Alzheimer’s disease by adding more information through other imaging techniques. Implementation of more modern deep learning architectures such as EfficientNet or Vision Transformers (ViTs) are also possible to enhance the accuracy level and further cut down the training period since they are efficient and highly scale up for images classification. Future work could focus on optimizing the model for real-time applications in clinical settings, where time efficiency and interpretability are critical for providing timely diagnoses.

## Conclusion

This research presented a new concept in classifying Alzheimer’s disease (AD) in four classes, namely mild-demented, moderate-demented, non-demented, and very mild-demented. Employing transfer learning and adjusting three pre-trained convolutional neural networks AlexNet, MobileNetV2, and GoogleNet, the classification accuracy was very high with the pre-trained AlexNet model with ADAM solver achieving the highest accuracy. This high accuracy suggests that our approach could facilitate earlier and more accurate diagnosis of Alzheimer’s disease, which is critical for timely intervention. Earlier detection of AD allows for prompt treatment and management strategies, potentially improving patient outcomes by slowing disease progression.

The outcomes of this research have substantial clinical implications. For example, very high classification accuracy in the earlier stages of dementia, such as Very-Mild-Demented and Mild-Demented, implies that such a model may be operationalized in the clinical settings to assist medical practitioners in making an accurate and timely diagnosis of Alzheimer’s disease prior to what is currently possible, thereby improving the treatment interventions offered. Whereas AD is at its most challenging to diagnose at incipient stages due to various factors, the present approach provides a consistent and dependable approach that minimizes the chances of misdiagnosis and helps in preventing and improving the treatment of various disorders, hence increasing the well-being of patients.

Our methodology, in addition to Alzheimer’s disease, seems to have a good purpose for additional medical conditions, mostly due to the characteristics of medical imaging. This is because transfer learning can easily incorporate any pre-trained models, which makes it possible for use even in other degenerative conditions likely Parkinson’s disease or even various other imaging techniques such as CT or PET scans. Further fine-tuning would be needed to adapt the models to the specifics of each condition and imaging modality, but the general methodology remains applicable across various healthcare diagnostics.

In relation to the current methodologies in the field, our model boasts remarkable benefits in terms of both precision and processing time. Because we employed transfer learning technique rather than training models afresh, we cut down operational expenses and yet comparable accuracy was retained. This not only guarantees less time consumption when making the models but also makes the incorporation of artificial intelligence in medicine less challenging to the physicians who have little computing power. Given the superior performance coupled with ease of use, it is conceivable that our model will perform better than the conventional machine learning models, which depend on feature engineering and large data sets. As we improve our approach, the next steps in the work will concentrate on cross-dataset generalization of the models, enhancing potential analysis of the models through explainable AI, and implementing real-time clinical deployment to increase diagnostic capabilities and enhance patient care.

## Electronic supplementary material

Below is the link to the electronic supplementary material.

Supplementary Material 1

Supplementary Material 2

Supplementary Material 3

## Data Availability

The dataset and simulation files used during the current study are available from the corresponding author upon request.

## Code availability

Current code version: V1.0, Permanent link to code/repository used for this code version: https://github.com/KainatSaher/Alzheimer/blob/main/task.m, Software code languages, tools and services used: Matlab, Compilation requirements, operating environments, and dependencies: Core i7-9850H Precision 5540, 32GB RAM, Microsoft Windows, Support email for questions: adnan@gachon.ac.kr and ara4013@qatar-med.cornell.edu.
