# Supplementary material for: A fine-tuned convolutional neural network model for accurate Alzheimer’s disease classification
Source: Sci Rep. 2025 Apr 4;15:11616. doi: 10.1038/s41598-025-86635-2 (PMC11971367; doi:10.1038/s41598-025-86635-2)
Supplement: Supplementary file 2 — Supplementary Material 2 [file 41598_2025_86635_MOESM2_ESM.doc]

STROBE Statement—checklist of items

|  | Item No | Response |
| --- | --- | --- |
| **Title and abstract** | 1 | Both title and abstract are added. |
| Introduction | | |
| Background/rationale | 2 | Added |
| Objectives | 3 | Added |
| Methods | | |
| Study design | 4 | Added |
| Setting | 5 | Added |
| Participants | 6 | (*a*) *Cohort study*— Not Applicable |
|  |  | (*b*) *Cohort study*— Not Applicable |
| Variables | 7 | Added |
| Data sources/ measurement | 8 | Added |
| Bias | 9 | Not Applicable |
| Study size | 10 | Described in article |
| Quantitative variables | 11 | Not Applicable |
| Statistical methods | 12 | Not Applicable |
|  |  | (*b*) Descried in article |
|  |  | (*c*) Added |
|  |  | (*d*) Not applicable |
|  |  | (*e*) Described in results |

Continued on next page

| Results | | |
| --- | --- | --- |
| Participants | 13 | (a) Added |
|  |  | (b) Added |
| Descriptive data | 14 | (a) Added |
|  |  | (b) Not Applicable |
|  |  | (c) Not Applicable |
| Outcome data | 15 | Not Applicable |
|  |  | Added in results |
|  |  | Added in results |
| Main results | 16 | (*a*) Added |
|  |  | (*b*) Added |
|  |  | (*c*) Not applicable |
| Other analyses | 17 | Added |
| Discussion | | |
| Key results | 18 | Added |
| Limitations | 19 | Added |
| Interpretation | 20 | Added |
| Generalisability | 21 | Added |
| Other information | | |
| Funding | 22 | Not applicable |
