# Supplementary material for: A fine-tuned convolutional neural network model for accurate Alzheimer’s disease classification
Source: Sci Rep. 2025 Apr 4;15:11616. doi: 10.1038/s41598-025-86635-2 (PMC11971367; doi:10.1038/s41598-025-86635-2)

**Grad-CAM visualizations for the axial, sagittal, and coronal planes**

**Correctly classified images in Axial slices:**


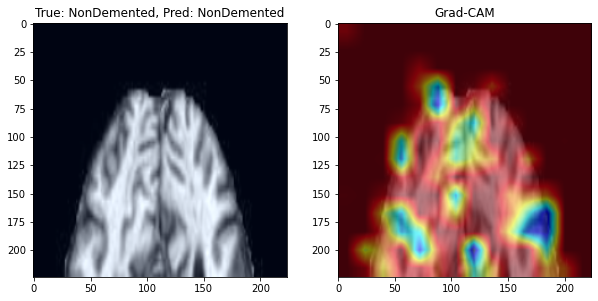


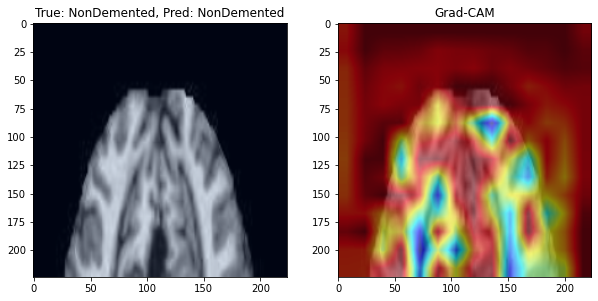


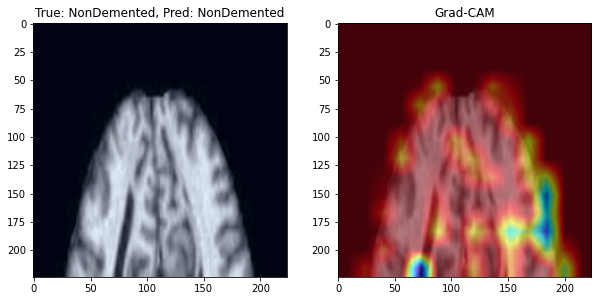


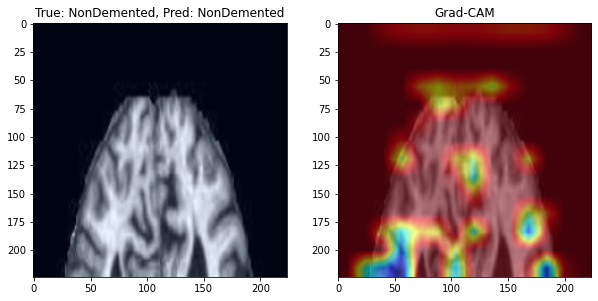


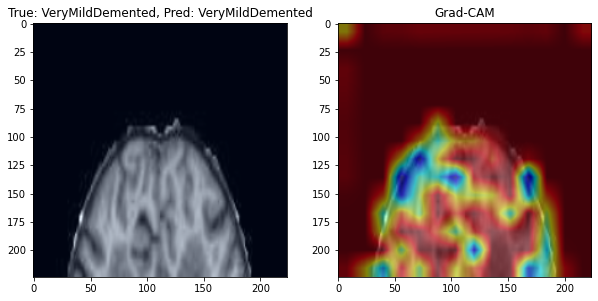


**Misclassified images in Axial slices:**


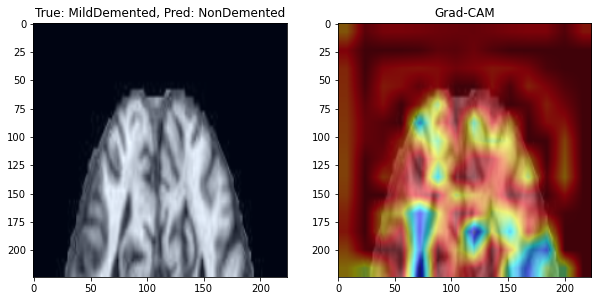


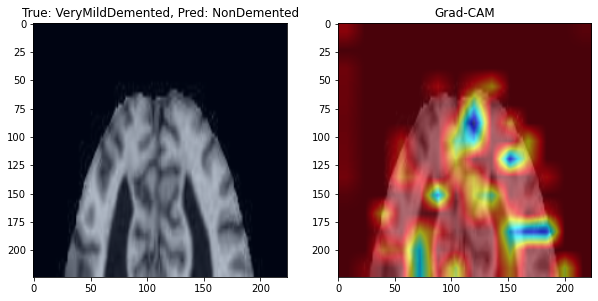


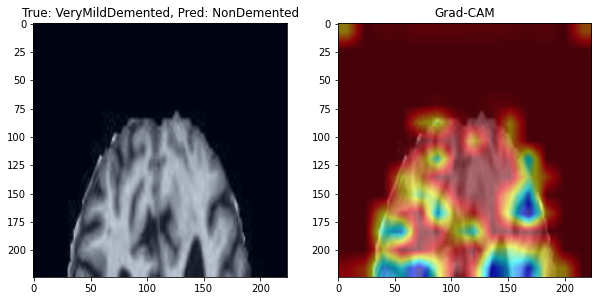


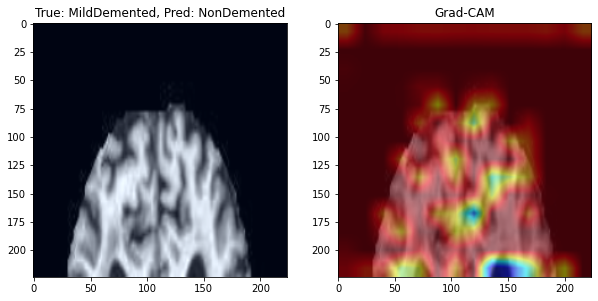


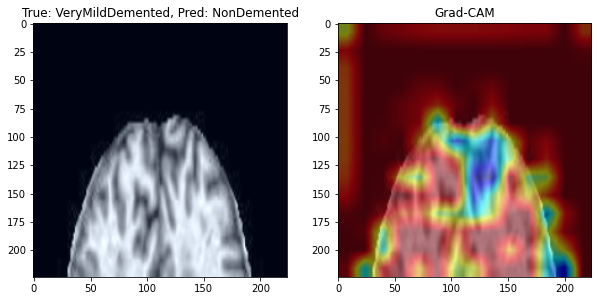


**Correctly classified images in Sagittal slices:**


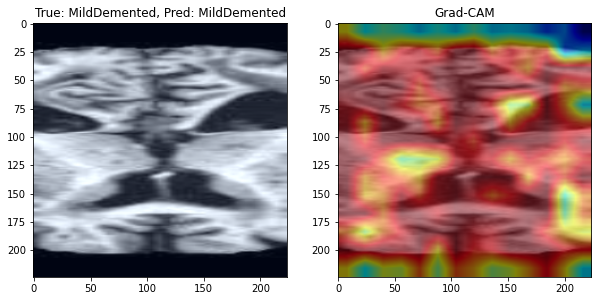


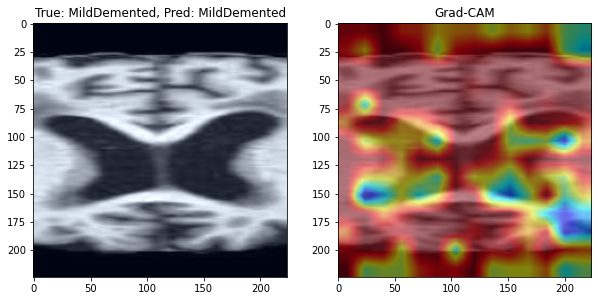


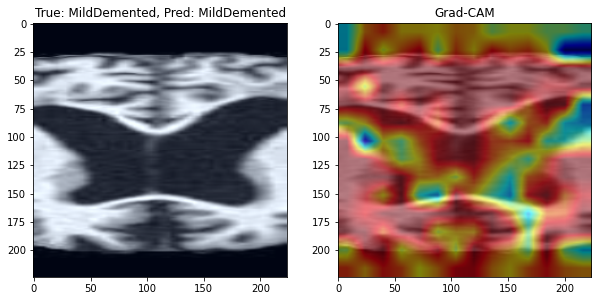


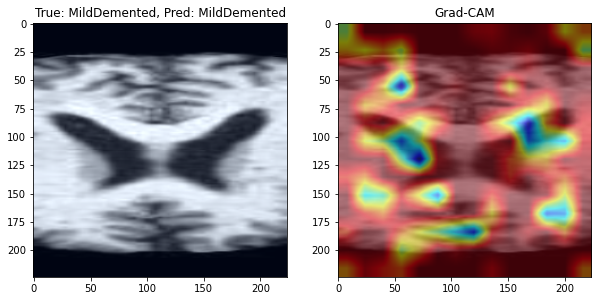


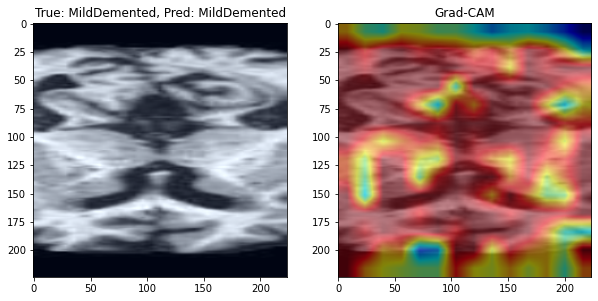


**Misclassified images in Sagittal slices:**


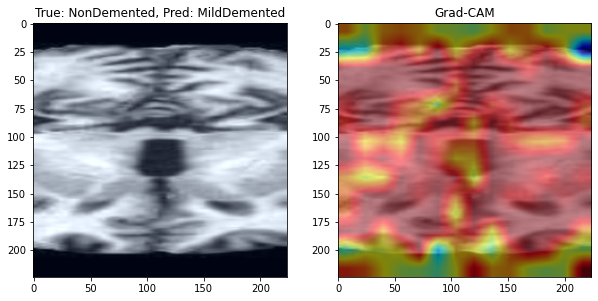


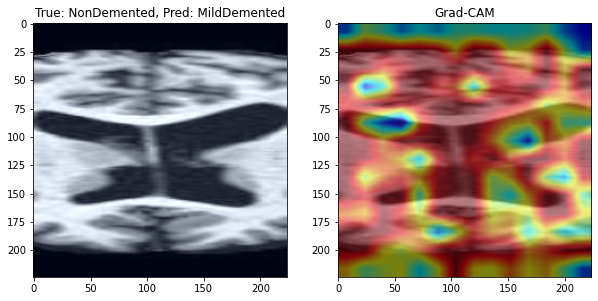


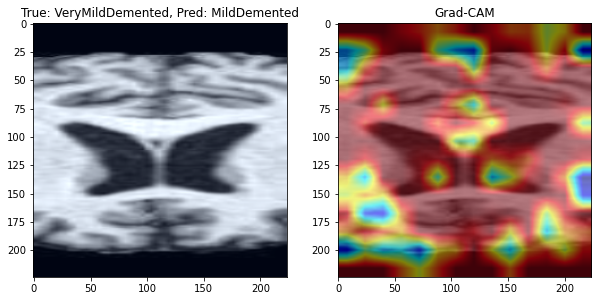


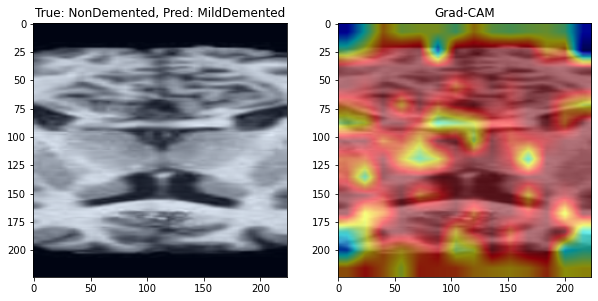


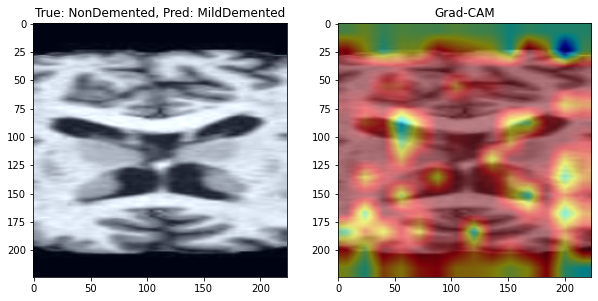


**Correctly classified images in Coronal slices:**


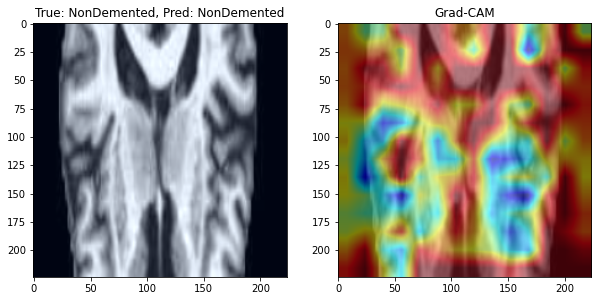


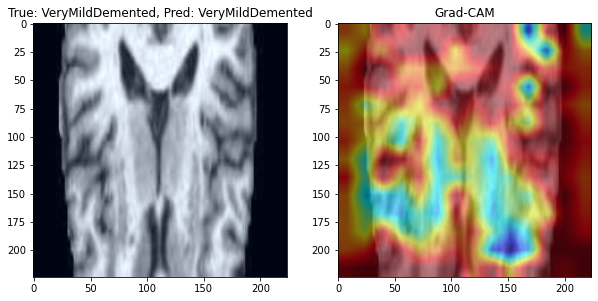


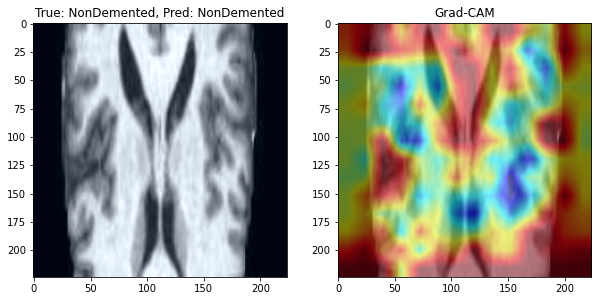


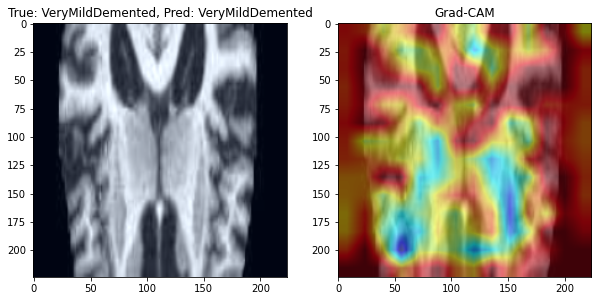


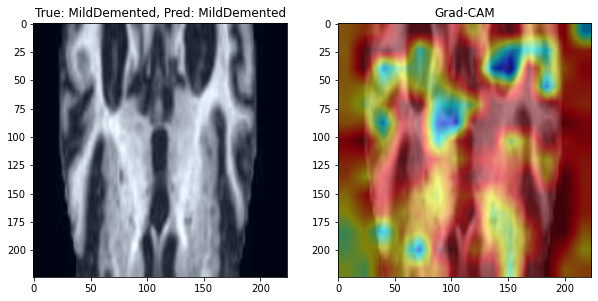


**Misclassified images in Coronal slices:**


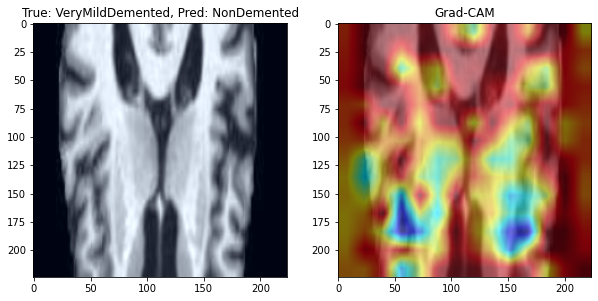


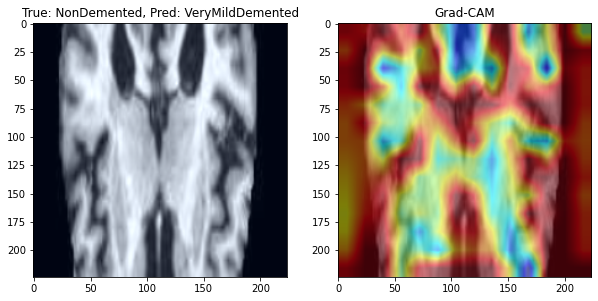


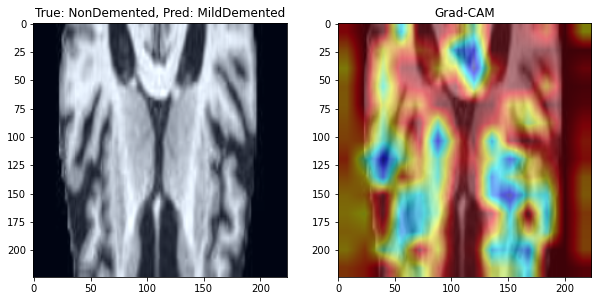


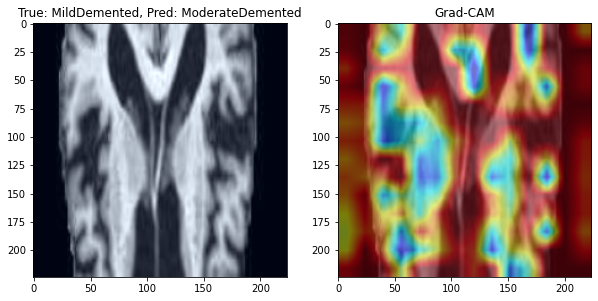


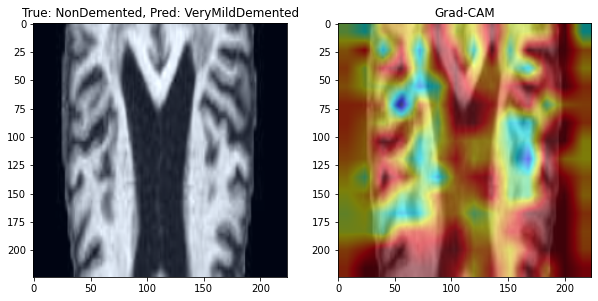

Supplement: Supplementary file 3 — Supplementary Material 3 [file 41598_2025_86635_MOESM3_ESM.docx]
